# Supplementary material for: Augmenting CAR T cell functionality and metabolism through CD39 downtuning
Source: Mol Ther Oncol. 2026 Jun 17;34(3):201277. doi: 10.1016/j.omton.2026.201277 (PMC13333332; doi:10.1016/j.omton.2026.201277)
Supplement: Document S1. Figures S1–S5 [file mmc1.pdf]

OMTON, Volume *34*

Supplemental information

Augmenting CAR T cell functionality

and metabolism through CD39 downtuning

Dennis Christoph Harrer, Jeremy Baldwin, Markus Barden, Hong Pan, Bence Gergely, Árpád Szöőr, György Vereb, Wolfgang Herr, and Hinrich Abken

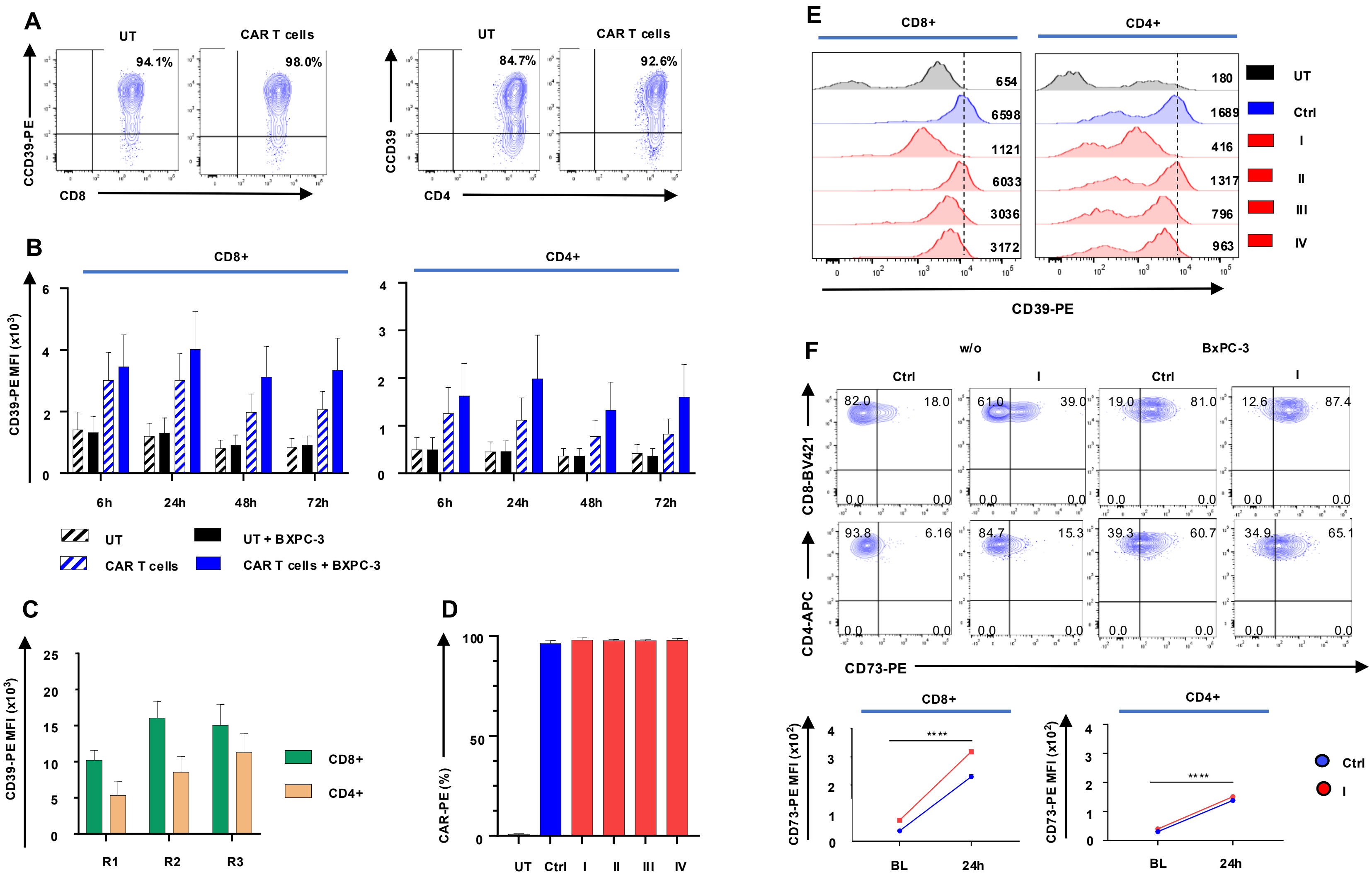

Figure S1: CD39 expression on CAR T cells. **(A)** CD39 expression on CD8⁺ and CD4⁺ CAR T cells was assessed by flow cytometry immediately after manufacturing (day 9 post-activation of PBMCs and retroviral transduction). Untransduced T cells (UT) served as controls. **(B)** Kinetics of CD39 expression on CD8⁺ and CD4⁺ CAR T cells following antigen-specific stimulation with CEA⁺ BxPC-3 pancreatic cancer cells, analyzed at the indicated time points post-stimulation. **(C)** CD39 expression during repetitive antigen stimulation. CAR T cells were co-cultured with CEA⁺ BxPC-3 cells over three stimulation rounds (R1–R3), each lasting three days. CD39 expression in CD8⁺ and CD4⁺ CAR T cells was analyzed by flow cytometry at the end of each round. **(D)** CEA-specific CAR T cells (CEA-28ζ-K = ctrl; CEA-28ζ-CD39-1 to CD39-4 = I–IV) were generated via retroviral transduction. UT cells were obtained by PBMC activation and expansion in IL-2 without CAR transduction. Following magnetic cell separation (MACS), CAR surface expression was assessed using a phycoerythrin (PE)-labeled goat anti-human IgG antibody. The percentage of CAR⁺ cells post-enrichment is shown. Data represent mean ± SEM of at least three donors. **(E)** Representative histograms showing CD39 expression (PE fluorescence) in CD8⁺ (left) and CD4⁺ (right) CAR T cells and UT cells, 24 hours after stimulation with CEA⁺ BxPC-3 cells. Mean fluorescence intensity (MFI) values for CD39-PE staining are indicated. Representative data from one of three donors are shown. **(F)** Representative dot plots showing CD73 expression in CD8⁺ and CD4⁺ CAR T cells (CEA-28ζ-K = Ctrl; CEA-28ζ-CD39-1 = I) 24 hours after stimulation with CEA⁺ BxPC-3 cells. Representative data from one of three donors are shown. Below, MFI of CD73-PE at baseline (BL) and 24 hours after antigen-specific stimulation with CEA⁺ BxPC-3 pancreatic cancer cells in CD8⁺ (left) and CD4⁺ (right) CAR T cells. Data represent mean ± SEM of three donors. Statistical significance was calculated using two-way ANOVA. ****$p \leq 0.0001$.

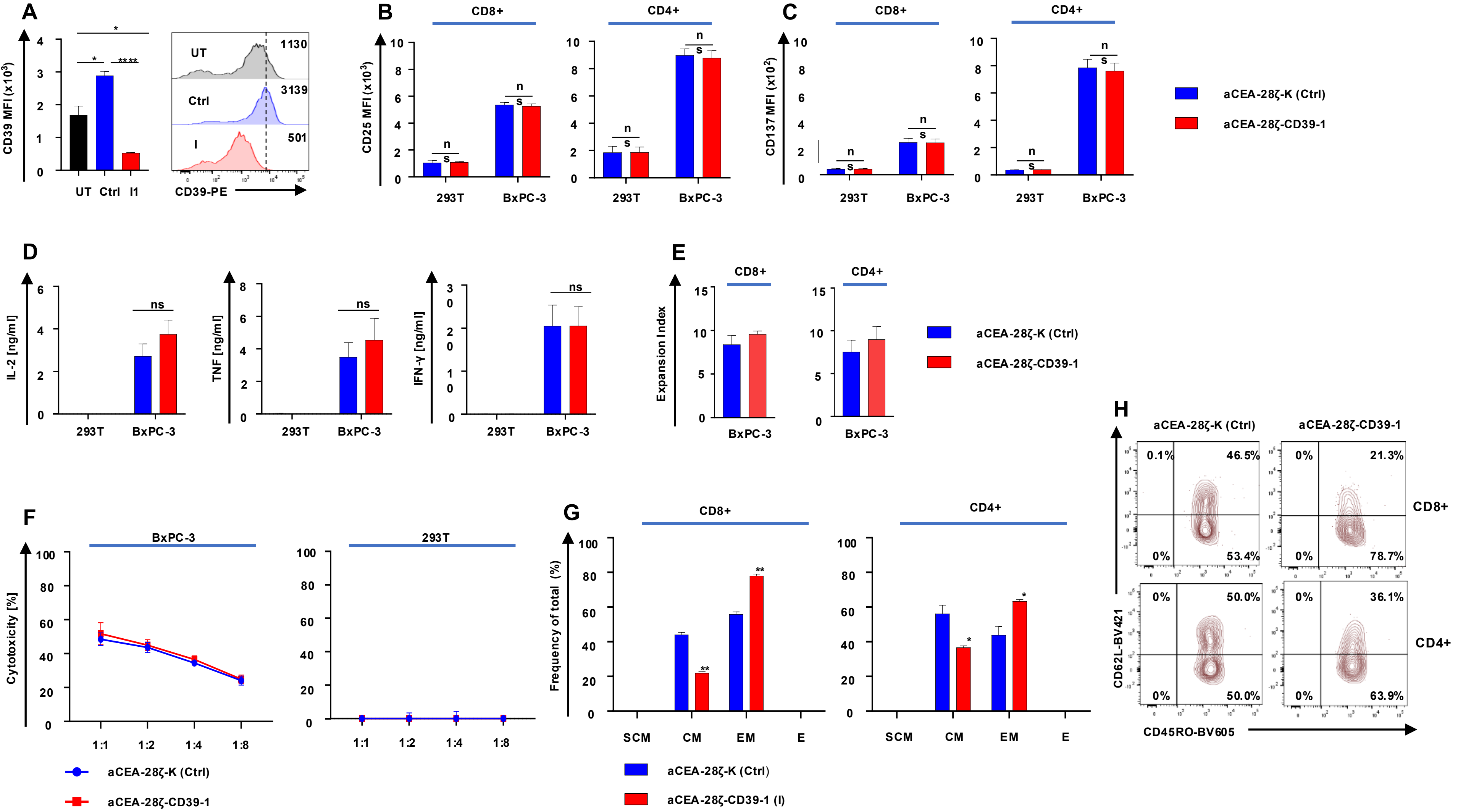

Figure S2: Impact of CD39 downregulation on basic CAR T cell effector functions. (A) CD39 expression in untransduced T cells (UT) and CAR T cells (CEA-28ζ-K = Ctrl; CEA-28ζ-CD39-1 = I) at the beginning of in vitro assays. CD39 expression was measured by flow cytometry, and mean fluorescence intensity (MFI) values of CD39-PE staining are shown in representative histograms. Data represent mean ± SEM of three donors. Statistical significance was determined using Student's t-test. *p ≤ 0.05; ***p ≤ 0.0001. (B) Antigen-specific upregulation of CD25 on CD8$^+$ and CD4$^+$ CAR T cells 24 hours after co-culture with CEA$^+$ BxPC-3 pancreatic cancer cells or control 293T cells. (C) Antigen-specific upregulation of CD137 (4-1BB) on CD8$^+$ and CD4$^+$ CAR T cells 24 hours after co-culture with BxPC-3 or 293T cells. (D) Cytokine secretion (IL-2, TNF-α, IFN-γ) by CAR T cells after 48-hour co-culture with BxPC-3 or 293T cells, measured by ELISA. (E) Proliferative capacity of CD8$^+$ and CD4$^+$ CAR T cells after five-day stimulation with BxPC-3 cells. T cells were labeled with Cell Proliferation Dye eFluor® 450, and the expansion index was calculated based on dye dilution. (B–E) Data represent mean ± SEM of three independent donors. Statistical analysis was performed using Student's t-test. ns = not significant. (F) Cytotoxic activity of CAR T cells following a 24-hour co-culture with BxPC-3 or 293T target cells at the indicated effector-to-target (E:T) ratios. Cytotoxicity was measured using an XTT-based colorimetric assay. Data represent the mean ± SEM of four donors. (G) Differentiation of CAR T cells into effector memory subsets after three rounds (R1–R3) of antigen-specific stimulation with unlabeled BxPC-3 cells. Memory subsets were defined as follows: T stem cell memory (SCM, CD45RO$^+$CD62L$^+$), central memory (CM, CD45RO$^+$CD62L$^+$), effector memory (EM, CD45RO$^+$CD62L$^-$), and effector (E, CD45RO$^-$CD62L$^-$) cells. Data represent the mean ± SEM of three donors. Statistical significance was assessed by paired t-test. *p ≤ 0.05; **p ≤ 0.01. (H) Representative dot plots corresponding to Figure 2 G (three donors).

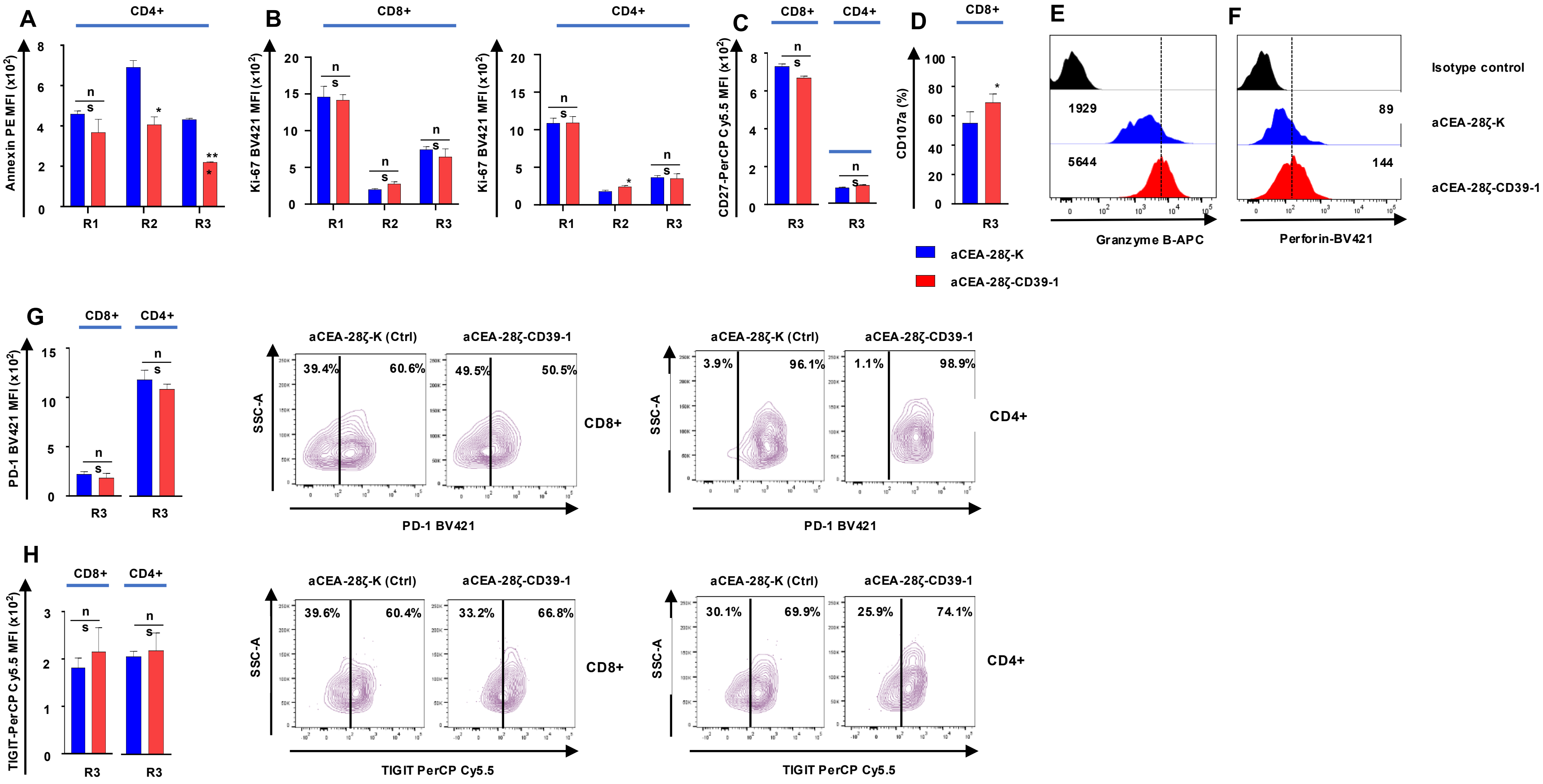

Figure S3: Phenotypic analysis of CAR T cells during repetitive antigen stimulation. CAR T cells (CEA-28ζ-K = ctrl; CEA-28ζ-CD39-1 = I) were stimulated three times (R1–R3) with unlabeled CEA⁺ BxPC-3 pancreatic cancer cells. Functional and phenotypic markers were evaluated at the end of each round as indicated. **(A)** Apoptosis in CD4⁺ CAR T cells was assessed at the end of each stimulation round by staining with Annexin V. **(B)** Proliferative capacity of CD8⁺ and CD4⁺ CAR T cells was measured by intracellular staining for Ki-67 at the end of each round. **(C)** Expression of CD27, a memory and survival marker, was evaluated in CD8⁺ and CD4⁺ CAR T cells at the end of round 3 (R3). **(D)** CD107a degranulation assay was performed on CD8⁺ CAR T cells at the end of round 3 to assess cytotoxic potential. T cells were co-cultured with unlabeled BxPC-3 cells for 4 hours, and CD107a surface expression was measured by flow cytometry. **(E, F)** Expression of granzyme B **(E)** and perforin **(F)** in CD8⁺ CAR T cells was analyzed at the end of round 3. Representative flow cytometry plots from one of three experiments are shown. **(G)** PD-1 expression in CD8⁺ and CD4⁺ CAR T cells at the end of round 3. **(H)** TIGIT expression in CD8⁺ and CD4⁺ CAR T cells at the end of round 3. **(A–D, G, H)** Data represent mean ± SEM of three independent donors or representative dot plots (three donors). Statistical analysis was performed using Student's t-test. *p ≤ 0.05; ns = not significant.

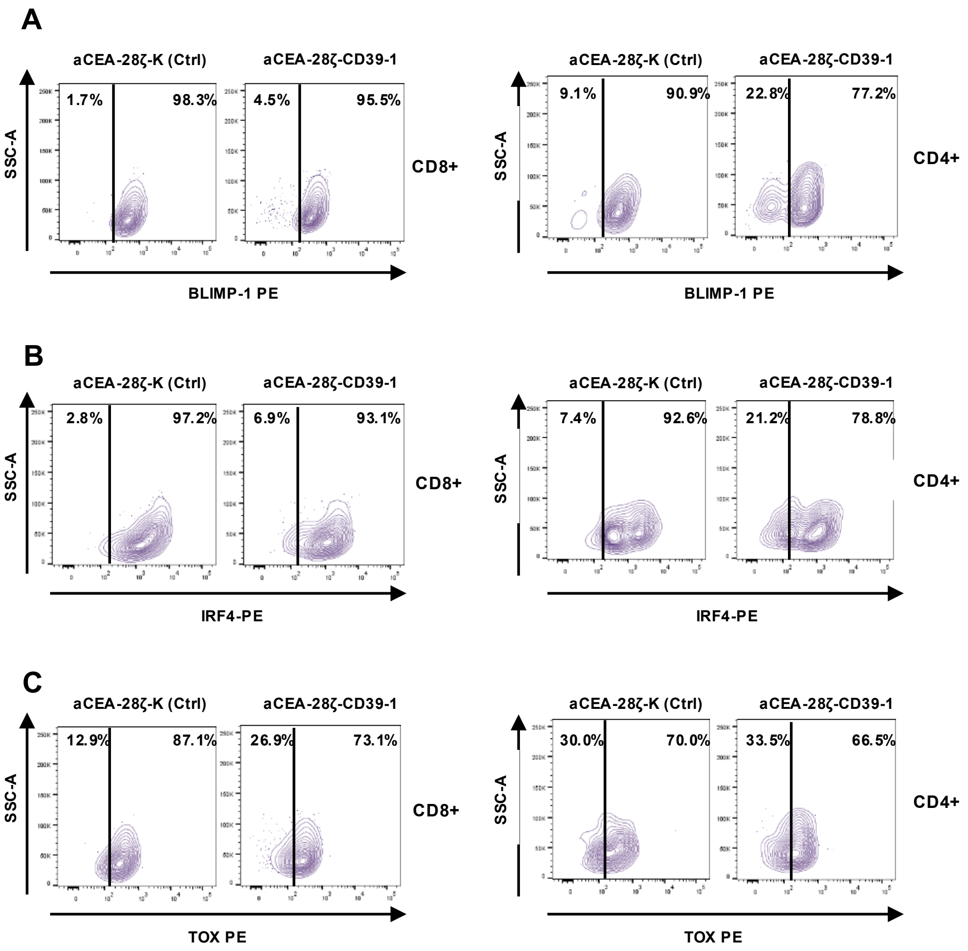

Figure S4: **Expression of exhaustion-related transcription factors in CAR T cells during repetitive antigen stimulation.** CAR T cells (CEA-28ζ-K = ctrl; CEA-28ζ-CD39-1 = I) were stimulated three times (R1–R3) with unlabeled CEA$^+$ BxPC-3 pancreatic cancer cells. Expression of BLIMP-1 **(A)**, IRF-4 **(B)**, and TOX **(C)** was determined via flow cytometry at the end of round three. One representative dot plot out of three donors is depicted.

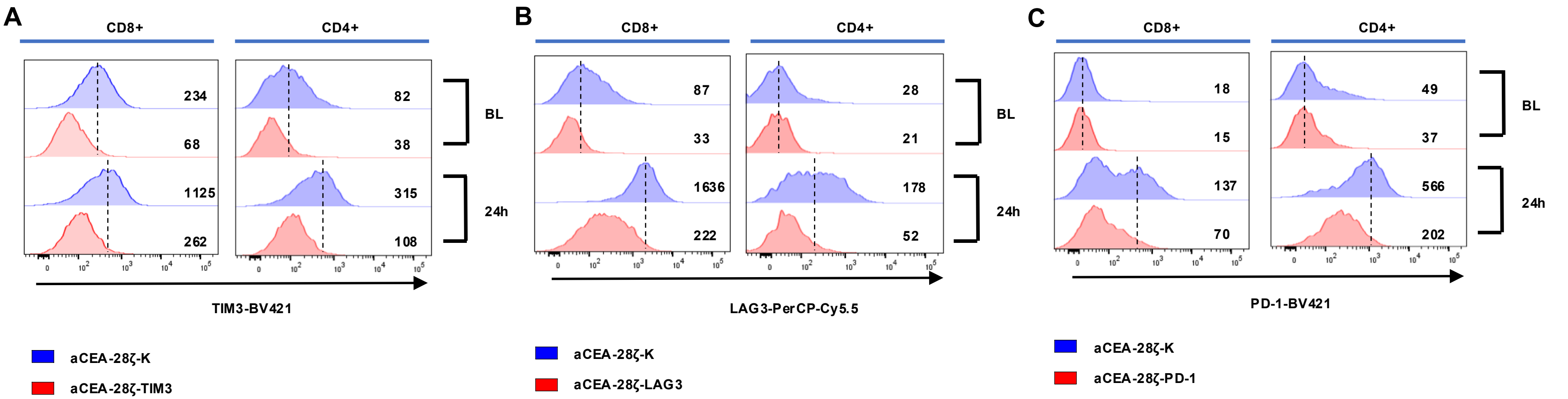

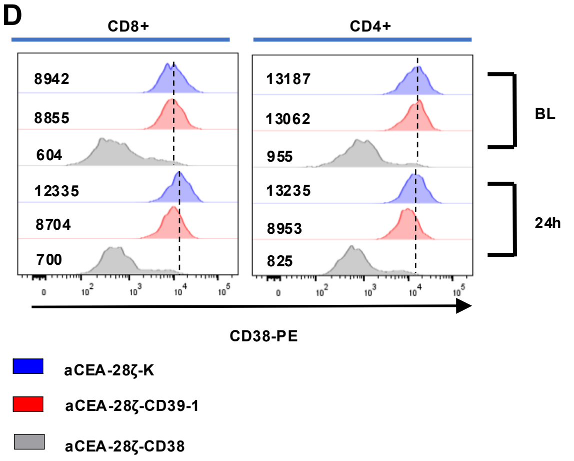

Figure S5: Downregulation of TIM-3, LAG-3, PD-1, and CD38 in CAR T Cells. (A–D) Representative histograms showing surface expression of inhibitory receptors at baseline (BL) and 24 hours after antigen-specific stimulation with CEA^{+} BxPC-3 pancreatic cancer cells in CD8^{+} (left) and CD4^{+} (right) CAR T cells. **(A)** TIM3**. (B)** LAG3**. (C)** PD-1. **(D)** CD38. Mean fluorescence intensity (MFI) values are indicated in each histogram. Data shown are from one representative donor out of three independent experiments.
