# Supplementary material for: Augmenting CAR T cell functionality and metabolism through CD39 downtuning
Source: Mol Ther Oncol. 2026 Jun 17;34(3):201277. doi: 10.1016/j.omton.2026.201277 (PMC13333332; doi:10.1016/j.omton.2026.201277)
Supplement: Document S2. Article plus supplemental information [file mmc2.pdf]

Molecular Therapy
Oncology
Original Article

Augmenting CAR T cell functionality and metabolism through CD39 downtuning

Dennis Christoph Harrer,[1,2] Jeremy Baldwin,[3,4] Markus Barden,[2] Hong Pan,[2] Bence Gergely,[5] Árpád Szöőr,[5] György Vereb,[5,6] Wolfgang Herr,[1] and Hinrich Abken[2]

[1]Department of Hematology and Internal Oncology, University Hospital Regensburg, 93053 Regensburg, Germany; [2]Leibniz Institute for Immunotherapy, Div. Genetic Immunotherapy, Chair Genetic Immunotherapy, University Regensburg, 93053 Regensburg, Germany; [3]Leibniz Institute for Immunotherapy, Div. Functional Immune Cell Modulation, University Regensburg, 93053 Regensburg, Germany; [4]Department of Pathology, University of Cambridge, Cambridge CB2 1QP, UK; [5]Department of Biophysics and Cell Biology, University of Debrecen Faculty of Medicine, 4032 Debrecen, Hungary; [6]University of Debrecen Faculty of Pharmacy, 4032 Debrecen, Hungary

The CD39/CD73 axis is a potent intrinsic repressor of T cell functionality. CAR T cell activation triggers the increase of CD39; its downregulation reduced extracellular ATP degradation and enhanced the functional capacities in CD39^{low} CAR T cells compared with conventional CAR T cells with respect to an increase in granzyme/perforin and degranulation upon repetitive CAR stimulation. CD39^{low} CAR T cells, moreover, displayed superior mitochondrial function and enhanced glycolytic activities. Consequently, CD39^{low} CAR T cells outperformed conventional CAR T cells in controlling CEA^{+} gastric carcinoma in xeno-transplanted NSG mice. The CD39 effect is unique, since downtuning CD38, also involved in the regulation of exhaustion, did not provide benefits under stimulatory "stress conditions". Activation-induced upregulation of CD39/CD73 contributes to a negative feedback loop for CAR T cells; downregulated CD39 levels augmented T cell anti-tumor activities by reducing AMP and adenosine-mediated repression. In the broader context, CD39 downregulation is unlikely to be sufficient as a standalone intervention in all settings but may be particularly valuable as part of combination strategies with complementary approaches targeting additional metabolic or immune checkpoint pathways to further enhance CAR T cell persistence and anti-tumor activity.

INTRODUCTION

Over the last decade, chimeric antigen receptor (CAR) T cells grew into a crucial pillar of tumor immunotherapy,[1] receiving regulatory approval for therapeutic use in patients with lymphomas and acute lymphoblastic leukemia.[2] A growing number of clinical trials are further investigating CAR T cell therapy in a wide variety of other malignancies.[3,4] Disappointingly, CAR T cells so far lack efficacy in patients with solid tumors; also, a substantial portion of patients with hematological malignancies suffer from relapses after initial tumor regression, making flexible adjustment of CAR T cell functionality necessary. Tremendous efforts are made in this direction to refine T cell maturation and persistence,[5] however, with limited success.

T cell dysfunctionality, commonly termed exhaustion, poses a significant intrinsic barrier to long-lasting CAR T cell efficacy against tumors.[6,7] Hallmarks of T cell exhaustion include effector cell differentiation, upregulation of inhibitory receptors, reduced proliferative capacity, absence of IL-2 production, as well as diminished cytotoxicity and release of pro-inflammatory cytokines.[8,9] Previous efforts to alleviate exhaustion-related CAR T cell dysfunctionality encompassed the knockdown of upregulated inhibitory molecules, such as PD-1,[10] TIM3,[11] LAG3,[12] CD38,[13] and TIGIT.[14] In contrast to those ligation-dependent inhibitory molecules, CD39 constitutes a ligand-independent inhibitor of T cell activity.[15] In light of the consistently elevated CD39 levels on tumor-reactive but terminally exhausted T cells,[16] CD39 has gained attention for being another mediator of T cell dysfunctionality.

CD39, also known as ectonucleotide-triphosphate-diphosphohydrolase-1 (ENTPD1), is an ectoenzyme on the surface of T cells that degrades adenosine triphosphate (ATP) into adenosine diphosphate (ADP). Subsequently, ADP is converted by the ectoenzyme CD73 via adenosine monophosphate (AMP) into adenosine, which impairs T cell functionality by binding to the immunosuppressive adenosine receptors A2A and A2B.[15] Under physiological conditions, the concentration of extracellular ATP is marginal; however, it can significantly rise in response to inflammation, hypoxia, necrosis, and cellular stress, all conditions particularly present in tumor tissues.[15] The CD39/CD73 axis exerts a 2-fold immunosuppressive effect on tumor-reactive T cells via (1) adenosine-induced suppression of T cell functionality and metabolism, and (2) degradation of extracellular ATP, which has been associated with impaired purinergic signaling, metabolic stress, and increased susceptibility to apoptosis.[17] Increased CD39 expression was frequently found on tumor-infiltrating CD8^{+} T cells serving as a reliable marker of terminal

Received 7 January 2026; accepted 15 June 2026;
https://doi.org/10.1016/j.omton.2026.201277.

Correspondence: Dennis Christoph Harrer, Department of Hematology and Internal Oncology, University Hospital Regensburg, 93053 Regensburg, Germany.
E-mail: dennis.harrer@ukr.de

Published by Elsevier Inc. on behalf of The American Society of Gene and Cell Therapy.
This is an open access article under the CC BY license (http://creativecommons.org/licenses/by/4.0/).

T cell exhaustion.[18] CD39^{high} T cells display diminished cytotoxicity, a reduced proliferative capacity, low production of inflammatory cytokines, and the expression of other inhibitory receptors like LAG3, TIGIT, PD1, and TIM3.[15,16,18,19] Overall, elevated CD39 levels on T cells are linked to poor survival in several types of cancer.[15] Collectively, these mechanisms give rise to our hypothesis that reducing CD39 levels on the CAR T cell surface may improve their functional capacities by reducing adenosine-mediated repression.

In this direction, deletion of CD39 in murine CD8$^+$ T cells enhanced anti-tumor functionality in murine melanoma models by impeding tumor progression, owing to an increased infiltration and functionality of tumor-specific T cells.[19] While each component of the degradation pathway from ATP to adenosine can be individually blocked, reducing CD39 activities confers the 2-fold advantage of simultaneously preserving ATP levels and reducing adenosine accumulation. In syngeneic tumor-bearing mice, CD39 inhibition alone delayed tumor growth more effectively than anti-CD73 antibodies or a small-molecule inhibitor of the adenosine A2A receptor.[15] Moreover, blocking CD39 by antibodies or pharmacological ectonucleotidase inhibitors could stunt tumor growth in several mouse models while simultaneously boosting the functionality of tumor-reactive T cells.[20] Anti-CD39 therapy was likewise effective in improving the functionality and tumor infiltration of terminally exhausted T cells resistant to anti-PD1 therapy.[21] Together, CD39 is a key regulator of T cell differentiation and metabolism, cytotoxicity, and cytokine secretion, which is also linked to coinhibitory receptors, such as PD-1.[21] For targeting CD39, three monoclonal antibodies have so far progressed to clinical evaluation in early phase trials for the treatment of patients with advanced solid tumors (NCT03884556, NCT04306900, and NCT04261075). Such CD39 targeting produces some benefits; however, treatment is associated with systemic side effects. We, therefore, aimed at restricting the CD39 blockade to the CAR T cell itself without affecting the overall physiological T cell response. Therewith, we reduced the levels of CD39 on CAR T cells through a specific shRNA that is expressed along with the CAR encoded by the same vector, restricting the functional capacities specifically to the CAR T cell.

Previous efforts to target the CD39/adenosine axis in CAR T cells have yielded mixed results. Klysz et al. demonstrated that CRISPR-mediated disruption of CD39 reduced extracellular adenosine accumulation and altered transcriptional programs associated with T cell differentiation[22]; however, only modest functional improvements were observed, prompting the quest for alternative metabolic rewiring approaches. Notably, these studies were performed primarily in a tonic-signaling exhaustion model in which CD39 expression was incompletely induced, and conventional CD19 CAR T cells showed minimal CD39 upregulation. More recently, Zhang et al. reported improved antitumor activity using CAR T cells engineered to secrete CD39-targeting nanobodies, although the relative contribution of tumor-cell versus CAR T cell targeting remained unclear.[23] Collectively, these studies suggest that modulation of CD39 can influence CAR T cell function, but that the magnitude and mechanisms of benefit may depend on the experimental context and mode of CD39 inhibition.

RESULTS

Downtuning CD39 in CAR T cells

Following TCR/CD3 stimulation and retroviral engineering, CAR T cells uniformly exhibited elevated CD39 levels (Figure S1A). Engagement of the CEA-specific CAR with its cognate antigen through incubation once or repeatedly with CEA$^+$ BxPC-3 pancreatic tumor cells further upregulated CD39 on CAR T cells (Figures S1B and S1C). Since high CD39 levels contribute to functional T cell repression through degradation of extracellular ATP to AMP, which is converted by CD73 into repressive adenosine, we proved our hypothesis that whether a reduction in CD39 levels prolongs CAR T cell activation. To attenuate CD39 expression, we employed a retroviral vector enabling co-expression of the CEA-specific CAR and a CD39-targeting shRNA, leveraging a previously established dual-expression vector system.[24] Four distinct shRNA constructs targeting CD39 (CEA-28ζ-CD39-1-4) were evaluated, alongside a control CAR vector containing a shRNA against the kanamycin resistance gene (CEA-28ζ-K) (Figure 1A).[25] All constructs yielded CAR levels in a similar range on T cells; CAR T cells were further enriched via magnetic-activated cell sorting (MACS; Figures 1B and S1D). Among the four constructs, CEA-28ζ-CD39-1 achieved the most efficient suppression of CD39 expression under antigen-directed stimulation with CEA$^+$ BxPC-3 cells (Figures 1C and S1E). This reduction was sustained across three consecutive rounds of antigen stimulation and was consistent in both CD8$^+$ and CD4$^+$ CAR T cell subsets (Figure 1D). CEA-28ζ-CD39-1 was used for all subsequent analyses; the other shRNAs had only modest effects. In sum, we successfully generated CD39^{low} CAR T cells using an integrated CAR-shRNA expression system, offering a streamlined, one-step manufacturing approach.

To test for CD39 ectoenzyme activities, we incubated CAR T cells with 200 μM ATP and monitored extracellular ATP degradation through ELISA. Compared with CEA-28ζ-K cells, CAR T cells with downregulated CD39 expression exhibited significantly reduced ATP degradation across all time points (Figure 1E). Conversely, the concentration of adenosine was significantly lower in CAR T cells with CD39 downregulation as compared to control CAR T cells (Figure 1F). Additionally, we assessed extracellular ATP release from CAR T cells following a 24-h CAR stimulation with a plate-bound anti-idiotypic antibody against the CAR or with CEA$^+$ BxPC3 cells. ATP release was detected in both T cells expressing the CEA-28ζ-K and CEA-28ζ-CD39-1 vectors. CAR T cells with downregulated CD39 displayed a greater accumulation of extracellular ATP in response to antigen-specific-activation relative to conventional CAR T cells (Figure 1G). The ectoenzyme CD73 is also upregulated by CAR T cells following antigen-specific stimulation (Figure S1F), which is of relevance due to its involvement in the signaling pathway downstream of CD39.

We assessed whether CD39 knockdown altered fundamental CAR T cell functions. Baseline CD39 expression was significantly lower

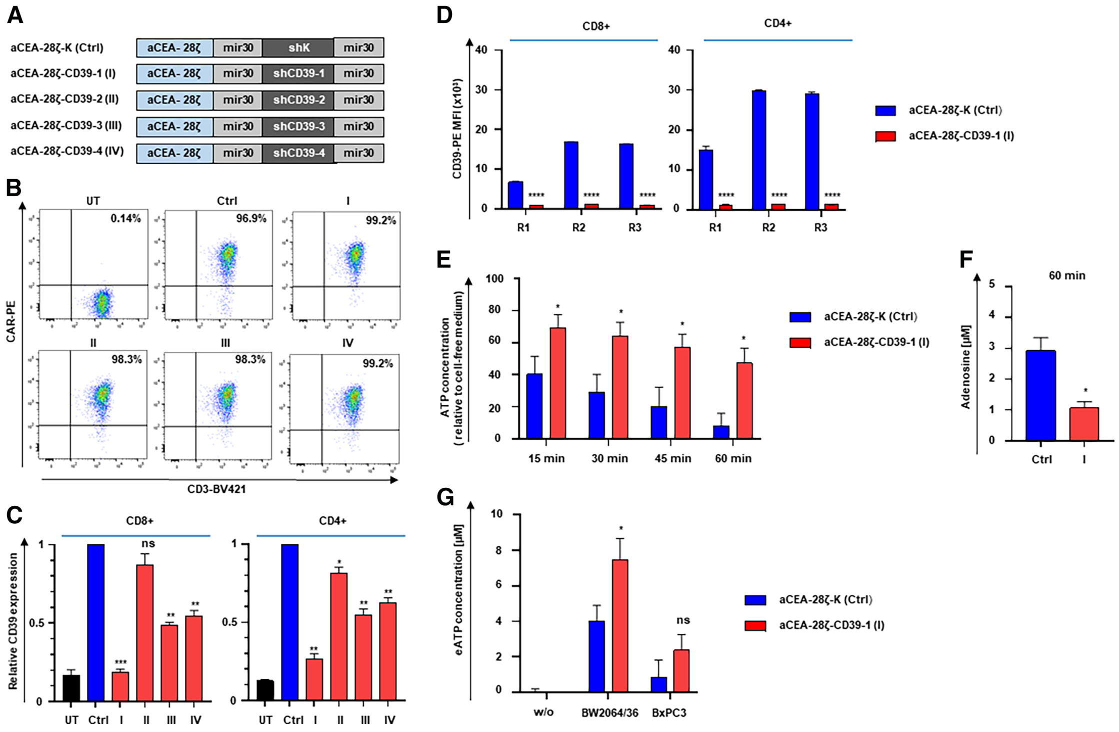

Figure 1. CD39 downtuning in CAR T cells

(A) Schematic representation of the CAR constructs used in the study. (B) CAR expression in T cells was assessed by staining with a phycoerythrin (PE)-labeled goat anti-human IgG antibody following magnetic-activated cell sorting (MACS). Untransduced T cells (UT) served as controls. Representative data from one of three donors are shown. (C) CD39 expression in $CD8^+$ (left) and $CD4^+$ (right) CAR T cells 24 h after stimulation with CEA^+ BxPC-3 cells. UT cells served as controls. Data represent the mean ± SEM of three donors. Statistical significance was assessed using paired *t* test. $^*p \leq 0.05$; $^{**}p \leq 0.01$; $^{***}p \leq 0.001$; ns, not significant. (D) CD39 expression in $CD8^+$ (left) and $CD4^+$ (right) CAR T cells during repetitive stimulation with CEA^+ BxPC-3 cells. Data represent the mean ± SEM of three donors. Statistical significance was determined using two-way ANOVA. $^{****}p \leq 0.0001$. (E) CAR T cells (2×10^5) were resuspended in 200 µL serum-free medium, and ATP was added to a final concentration of 200 µM. At the indicated time points (0 min = starting point), supernatants were collected, and extracellular ATP concentrations were measured by ELISA and normalized to the cell-free medium control (set to 1). Data are expressed as the mean ± SEM from three donors. Statistical significance was assessed using two-way ANOVA. $^*p \leq 0.05$. (F) CAR T cells (2×10^5) were resuspended in 200 µL serum-free medium, and ATP was added to a final concentration of 200 µM. After 60 min, supernatants were collected, and extracellular adenosine concentrations were measured by ELISA. Data are expressed as the mean ± SEM from fours donors. Statistical significance was assessed using paired *t* test. $^*p \leq 0.05$. (G) CAR T cells were incubated for 24 h either in medium alone (w/o), on a plate coated with the anti-idiotypic antibody BW 2064/36 (1.5 µg/mL, stimulates CEA CAR), or with BxPC3 cells. After incubation, supernatants were collected, and extracellular ATP concentrations were measured by ELISA. Data are presented as the mean ± SEM from five donors. Statistical significance was determined using two-way ANOVA. $^*p \leq 0.05$, ns = not significant.

in CEA-28ζ-CD39-1 CAR T cells compared with controls (Figure S2A). Antigen-driven upregulation of activation markers CD25 and CD137 in both $CD8^+$ and $CD4^+$ CAR T cells was not affected by CD39 downregulation (Figures S2B and S2C). Additionally, secretion of pro-inflammatory cytokines, such as IL-2, TNF-α, and IFN-γ, in response to BxPC-3 cell engagement was similar between the two groups (Figure S2D), as was antigen-driven proliferation (Figure S2E). In a cytotoxicity assay, both CEA-28ζ-CD39-1 and control CEA-28ζ-K CAR T cells exhibited similar antigen-specific killing of CEA^+ BxPC-3 cells across a range of effector-to-target ratios, with negligible off-target cytotoxicity against CEA- 293T cells (Figure S2F). Phenotypically, repetitive antigen exposure of CD39 downregulated CAR T cells and promoted their effector memory differentiation (Figures S2G and S2H). In aggregate, efficient and durable downregulation of CD39 was attained in engineered CAR T cells which, importantly, did not impair key effector capacities.

CD39 downtuning improves CAR T cell functionality under conditions of repetitive stimulation *in vitro* and in the tumor control *in vivo*

To assess the long-term functional persistence of CAR T cells with reduced CD39 levels, we employed a "stress-test" involving repetitive antigen challenges *in vitro*.[24,26] CEA-28ζ-CD39-1 CAR T cells with CD39 knockdown underwent initial expansion followed by a contraction phase, ultimately resulting in a higher number of surviving CAR T cells compared with control CEA-28ζ-K CAR T cells (Figure 2A). Remarkably, in the third round of antigen exposure, CEA-28ζ-CD39-1 CAR T cells retained the ability to eliminate approximately 50% of seeded BxPC-3 target cells, whereas the

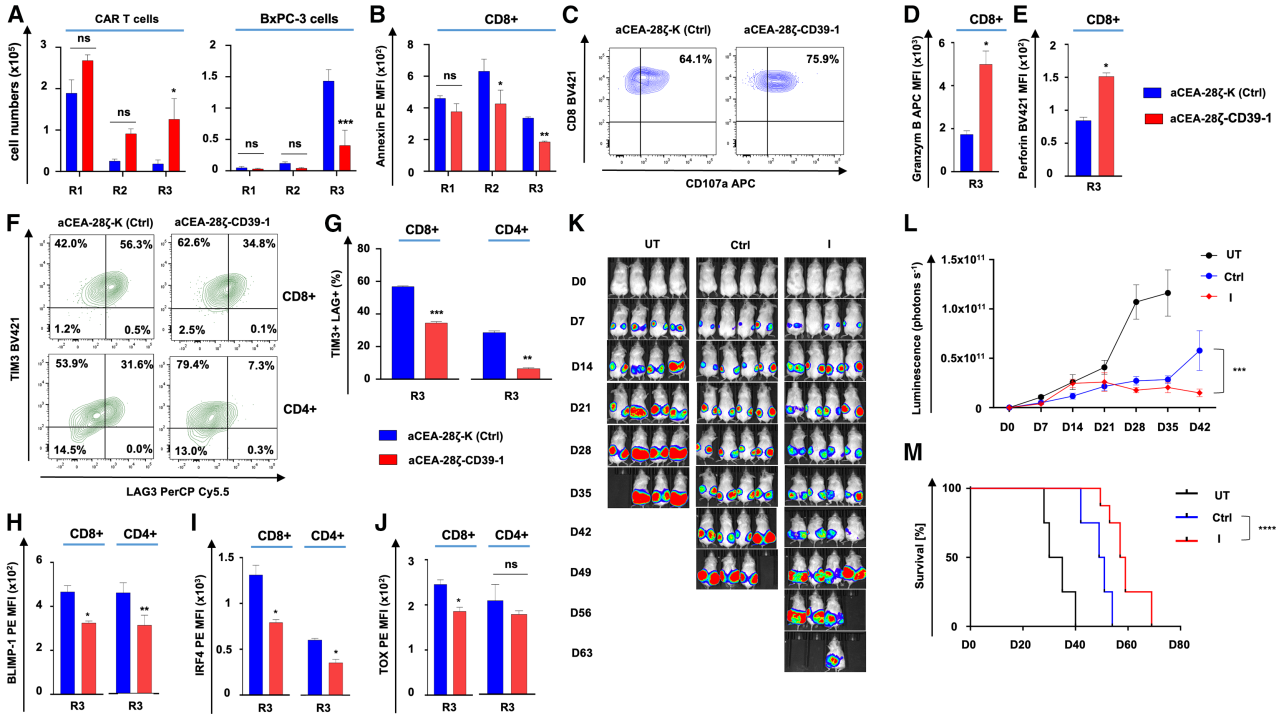

Figure 2. CD39 downtuning improves CAR T cell functionality

(A) CAR T cells (CEA-28ζ-K as control; CEA-28ζ-CD39-1) (1×10^5 CAR T cells) were stimulated over three consecutive rounds (R1–R3) with GFP-labeled CEA$^+$ BxPC-3 tumor cells (1×10^5 cells per round). At the end of each round, CAR T cells (live CD3$^+$ CAR$^+$; left) and residual tumor cells (live GFP$^+$; right) were quantified via flow cytometry. Data represent mean ± SEM of four donors. Statistical significance was assessed by two-way ANOVA. $^*p \leq 0.05$; $^{****}p \leq 0.0001$; ns, not significant. (B) CD8$^+$ CAR T cells were assessed for apoptosis using Annexin V staining after each stimulation round (R1–R3) with unlabeled BxPC-3 cells. (C and D) Expression of granzyme B (C) and perforin (D) in CD8$^+$ CAR T cells following three rounds (R3) of antigen stimulation. (B–D) Data represent mean ± SEM of three donors. Statistical significance was determined using paired *t* test. $^*p \leq 0.05$; $^{**}p \leq 0.01$; ns, not significant. (E) CD107a degranulation assay was performed to evaluate the cytotoxic potential of CD8$^+$ CAR T cells after the third round of stimulation. CAR T cells were co-incubated with unlabeled BxPC-3 cells for 4 h, followed by surface staining for CD107a. One representative experiment out of three is shown. (F–J) Phenotypic profiling of CAR T cells (CEA-28ζ-K as control; CEA-28ζ-CD39-1) after three rounds (R3) of antigen stimulation with unlabeled BxPC-3 cells. Expression of the following markers was analyzed by flow cytometry: LAG-3 and TIM-3 (F and G), BLIMP-1 (H), IRF4 (I), and TOX (J). Data represent mean ± SEM or representative dot plots of three donors. Paired *t* tests were used for statistical analysis. $^*p \leq 0.05$; $^{**}p \leq 0.01$; ns, not significant. (K–M) *In vivo* antitumor activity of CD39-downtuned CAR T cells. NSG mice were subcutaneously inoculated with 1×10^6 CEA$^+$ N87 gastric cancer cells expressing firefly luciferase. On day 10, mice received 0.25×10^6 CAR T cells (CEA-28ζ-K as control; CEA-28ζ-CD39-1) via intravenous injection. UT, untreated control group (no T cell transfer). (K) Tumor progression was monitored weekly via bioluminescence imaging in groups of 4 mice per condition (8 tumors per group). (L) Quantification of tumor burden over time based on luminescence intensity (photons/s/cm^2/sr). Data represent mean ± SD. Statistical significance was calculated using two-way ANOVA. $^{***}p \leq 0.001$. (M) Kaplan-Meier survival curves were generated based on tumor-level endpoints (8 tumors per group). Each mouse carried two subcutaneous tumors (one per flank). Tumor-specific events were analyzed individually, while humane endpoint criteria requiring euthanasia of an animal resulted in simultaneous censoring of both tumors from that mouse. This experimental design was performed in accordance with the 3Rs principles to reduce the number of animals used while allowing assessment of treatment effects across two tumor sites per biological replicate. Survival curves were compared using the log rank (Mantel-Cox) test. $^{****}p \leq 0.0001$.

cytotoxic activity of the canonical CEA-28ζ-K CAR T cells was nearly abolished (Figure 2A).

Mechanistically, CD39 downregulation conferred resistance to apoptosis in later stages of repeated antigen stimulation, as evidenced by significantly lower levels of apoptosis in CEA-28ζ-CD39-1 CAR T cells compared with controls (Figures 2B and S3A). Despite the differences in survival, both groups exhibited similar proliferative capacities (Figure S3B). The expression of the memory-associated marker CD27 remained similar between both CAR T cell populations (Figure S3C). Functionally, CD8$^+$ CEA-28ζ-CD39-1 CAR T cells demonstrated enhanced cytotoxic activity at the end of round three, as indicated by increased degranulation (Figures 2C and S3D) and elevated levels of granzyme B (Figures 2D and S3E) and perforin (Figures 2E and S3F). The enhanced functionality was accompanied by exhaustion-related LAG-3 and TIM-3 double-positive CAR T cells (Figures 2F and 2G), while PD-1 and TIGIT levels were not altered (Figures S3G and S3H). In this line, CEA-28ζ-CD39-1 CAR T cells expressed lower levels of the key exhaustion-related transcription factors, BLIMP-1, IRF4, and TOX (Figures 2H, 2J, and S4A–S4C), as revealed by flow cytometry. Data indicate a less exhausted phenotype of CAR T cells with down tuned CD39 under chronic stimulatory conditions.

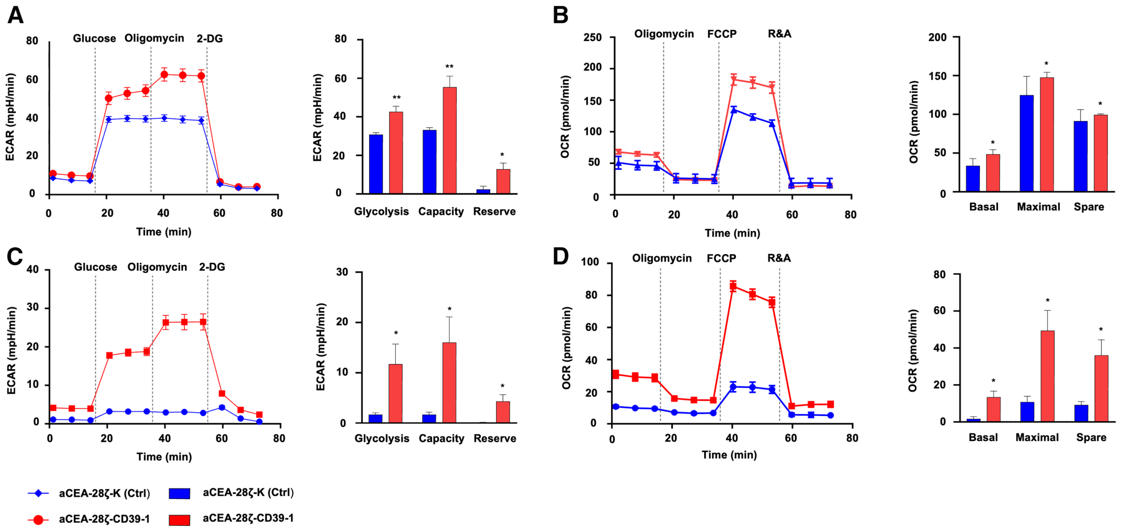

Figure 3. CD39 downtuning improves metabolic capacities of CAR T cells

Metabolic profiling of CAR T cells (CEA-28ζ-K as control; CEA-28ζ-CD39-1) was performed using a Seahorse extracellular flux assay. (A) Baseline extracellular acidification rate (ECAR), indicative of glycolytic activity. 2-DG, 2-deoxy-D-glucose. (B) Baseline oxygen consumption rate (OCR), indicative of mitochondrial respiration. FCCP, carbonyl cyanide-4-(trifluoromethoxy) phenylhydrazone; R&A, rotenone and antimycin A. (C and D) CAR T cells were subjected to three rounds (R1–R3) of stimulation with CEA$^+$ BxPC-3 cells. At the end of round 3, CAR T cells were isolated via magnetic-activated cell sorting (MACS), and metabolic activity was assessed by measuring (C) ECAR and (D) OCR. In (A)–(D), data represent mean ± SEM of at least four donors, with a minimum of three technical replicates per condition. Statistical significance was determined using Student's *t* test. *$p \leq 0.05$; **$p \leq 0.01$; ns, not significant.

We also evaluated the therapeutic efficacy of CAR T cells in the long-term control of tumors *in vivo*. In NSG mice inoculated with CEA$^+$ N87 gastric cancer cells, CEA-28ζ-CD39-1 CAR T cells achieved superior tumor control and significantly improved survival of tumor-bearing animals compared with canonical CAR T cells with physiological CD39 levels (Figures 2K–2M). In sum, CD39 downtuning significantly improved CAR T cell performance under repetitive antigen exposure and conferred superior CAR T cell performance *in vivo*.

CD39 downtuning refines glycolytic capacity and mitochondrial function in CAR T cells

To further elucidate the mechanistic basis for the enhanced functional capacities observed in CD39-downregulated CAR T cells, we assessed their metabolic profiles using a Seahorse assay. Notably, even prior to repetitive antigen stimulation, CEA-28ζ-CD39-1 CAR T cells demonstrated a significantly higher glycolytic capacity compared with control CEA-28ζ-K CAR T cells (Figure 3A), alongside improved mitochondrial function (Figure 3B). These findings suggest that CD39 downregulation may influence the metabolic state of CAR T cells already at baseline, in addition to its role during chronic antigen exposure. To evaluate metabolic fitness under chronic antigen exposure, CAR T cells were subjected to three rounds of stimulation with CEA$^+$ BxPC-3 target cells. Post-stimulation, CAR T cells were purified by MACS and analyzed for metabolic activities. Following repetitive antigen challenge, CEA-28ζ-CD39-1 CAR T cells maintained robust glycolytic activity and mitochondrial function, whereas these metabolic capacities were nearly extinguished in conventional CEA-28ζ-K CAR T cells (Figures 3C and 3D). These findings corroborate the results from the *in vitro* "stress-test," supporting the notion that CD39 downregulation preserves T cell metabolic functions under chronic CAR stimulation. Thus, enhanced metabolic fitness likely represents a key mechanistic basis for the superior persistence and functionality observed in CD39-downtuned CAR T cells.

CD39 downtuning is superior in anti-cancer cell efficacy over TIM3, LAG3, PD-1, or CD38 downregulation in CAR T cells

To systematically compare the functional impact of CD39 downregulation with that of blocking inhibitory receptors or metabolic checkpoints, we engineered retroviral constructs enabling co-expression of a CEA-specific CAR (CEA-28ζ) and shRNA-mediated knockdown of either PD-1, TIM3, LAG3, or CD38 (Figure 4A). All CAR constructs produced similar CAR levels in transduced T cells (Figure 4B). Notably, PD-1 expression was predominantly in CD4$^+$ CAR T cells, whereas TIM3 and LAG3 were more highly expressed in CD8$^+$ CAR T cells. Upon antigen-specific stimulation with BxPC3 tumor cells, expression of the targeted inhibitory receptors was efficiently suppressed in their respective CAR T cell products (TIM3 in CEA-28ζ-TIM3, LAG3 in CEA-28ζ-LAG3, and PD-1 in CEA-28ζ-PD-1 CAR T cells), confirming effective expression silencing (Figures 4C–4E and S5A–S5C). Knockdown of PD-1, TIM3, or LAG3 did not confer a functional advantage over control CEA-28ζ-K CAR T cells (Figure 4F); this is in clear contrast with CD39 downregulation, which significantly enhanced the

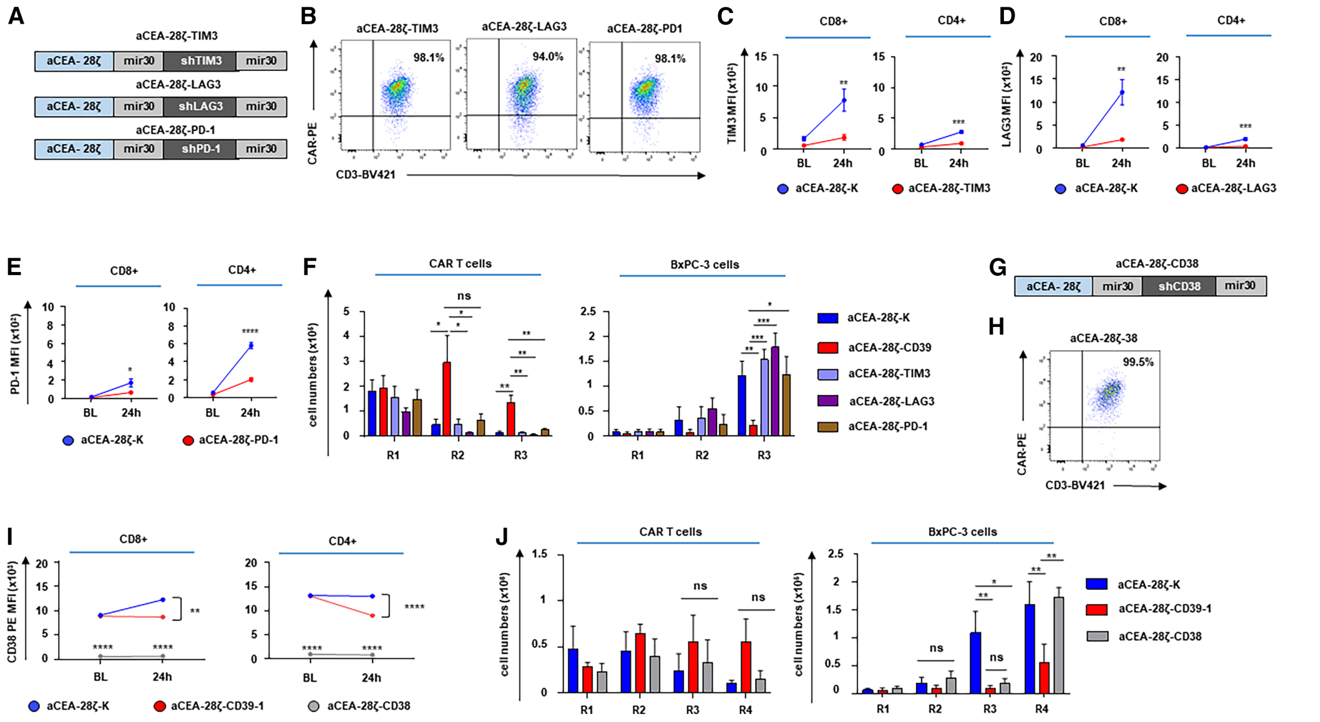

Figure 4. CD39 downtuning enhances CAR T cell functionality relative to downregulation of TIM-3, LAG-3, PD-1, or CD38 in a repetitive antigen stimulation assay

(A) Schematic representation of the CAR constructs. (B) CAR surface expression in T cells transduced with constructs targeting TIM-3 (CEA-28ζ-TIM3), LAG-3 (CEA-28ζ-LAG3), or PD-1 (CEA-28ζ-PD1). Representative flow cytometry data from one of three donors are shown. (C–E) Verification of knockdown efficiency for inhibitory receptors in CD8^{+} and CD4^{+} CAR T cells at baseline (BL) and after 24 h of stimulation with CEA^{+} BxPC-3 cells. (C) TIM-3 downregulation in CEA-28ζ-TIM3 CAR T cells. (D) LAG-3 downregulation in CEA-28ζ-LAG3 CAR T cells. (E) PD-1 downregulation in CEA-28ζ-PD1 CAR T cells. Data represent mean ± SEM from three donors. Statistical analysis was performed using two-way ANOVA. *$p \leq 0.05$; **$p \leq 0.01$; ***$p \leq 0.001$; ****$p \leq 0.0001$. (F) Functional comparison of CAR T cells with different checkpoint modulations (CEA-28ζ-K as control; CEA-28ζ-CD39-1; CEA-28ζ-TIM3; CEA-28ζ-PD1; CEA-28ζ-LAG3) in a repetitive antigen stimulation assay. CAR T cells (1×10^{5}) were co-cultured with GFP-labeled CEA^{+} BxPC-3 tumor cells (1×10^{5}) over three rounds (R1–R3) of antigen stimulation. At the end of each round, CAR T cells (live CD3^{+} CAR^{+}; left) and tumor cells (live GFP^{+}; right) were quantified via flow cytometry. Data represent mean ± SEM from six donors. Statistical significance was assessed using two-way ANOVA. *$p \leq 0.05$; **$p \leq 0.01$; ***$p \leq 0.001$; ns, not significant. (G) Schematic of the CAR construct mediating CD38 downregulation (CEA-28ζ-CD38). (H) Surface expression of the CAR in CEA-28ζ-CD38 CAR T cells. One representative experiment out of three donors is shown. (I) CD38 downregulation in CD8^{+} and CD4^{+} CEA-28ζ-CD38 CAR T cells at baseline and after 24 h stimulation with BxPC-3 cells. Data represent mean ± SEM from three donors. Statistical analysis was performed using two-way ANOVA. **$p \leq 0.01$; ***$p \leq 0.001$; ****$p \leq 0.0001$. (J) Functional performance of CEA-28ζ-K (control), CEA-28ζ-CD39-1, and CEA-28ζ-CD38 CAR T cells in a repetitive stimulation assay over four rounds (R1–R4) with GFP-labeled CEA^{+} BxPC-3 cells. Quantification of CAR T cells (live CD3^{+} CAR^{+}; left) and tumor cells (live GFP^{+}; right) was performed at the end of each round via flow cytometry. Data represent mean ± SEM of four donors. Statistical significance was calculated using two-way ANOVA. *$p \leq 0.05$; **$p \leq 0.01$; ***$p \leq 0.001$; ns, not significant.

long-term cytotoxic function of CAR T cells under conditions of repetitive stimulation with cancer cells *in vitro* (Figure 4F).

Next, we asked whether downtuning CD38, which emerged as another activation-induced metabolic checkpoint implicated in T cell exhaustion,[13] likewise exhibits a protective capacity as does CD39. Therefore, we leveraged our CAR-shRNA platform to generate CEA-28ζ-CD38 CAR T cells for evaluation in the *in vitro* stress test (Figures 4G and 4H). These cells exhibited near-complete loss of CD38 expression both at baseline and after stimulation with BxPC3 cells (Figures 4I and S5D). Functionally, CD38-downregulated CAR T cells outperformed conventional CEA-28ζ-K CAR T cells during the third round of antigen re-stimulation, maintaining higher cytotoxic activity. However, following a fourth re-stimulation (R4), CEA-28ζ-CD38 CAR T cells lost cytotoxic capacity, indicating the onset of T cell dysfunction (Figure 4J). In contrast, CEA-28ζ-CD39 CAR T cells maintained sustained anti-tumor activity in R4 as well (Figure 4J). Collectively, the results demonstrate that specifically CD39 downregulation confers superior CAR T cell functionality compared with knockdown of PD-1, TIM3, LAG3, or CD38 under conditions of repetitive antigen stimulation, thus highlighting CD39 as a relevant metabolic target to prolong CAR T cell efficacy.

DISCUSSION

CD39 is attracting growing interest as a crucial factor in driving T cells into dysfunctionality, which is still the major hurdle for the

success of cancer immunotherapy of solid cancers.[18] To bolster the power of CAR T cell activity, we decreased the CD39 levels in CAR T cells by transgenic expression of a CD39-specific shRNA. To allow a one-step manufacturing procedure, we relied on our previously reported retroviral vector architecture that allows for the expression of both a shRNA module, targeting the gene of interest, and the CEA-specific CAR.[24] The vector achieved a robust CD39 knockdown, i.e., the CD39 levels were reduced by approximately 90% in CAR T cells, which stayed at background levels even after repetitive CAR stimulation. CD39 downtuning did not affect canonical T cell effector functions *in vitro*. Upon repetitive stimulation with cognate cancer cells, CD39-downtuned CAR T cells showed superior functional persistence and CAR-redirected cancer cell elimination as compared with conventional CAR T cells. Remarkably, engineered CD39^{low} CAR T cells displayed a lower frequency of apoptosis throughout the entire course of repetitive antigen challenges. Furthermore, CD39^{low} CAR T cells showed a less exhaustive phenotype characterized by the diminished expression of LAG3 and TIM3. The protection from entry into exhaustion becomes visible by reduced levels of BLIMP-1, IRF4, and TOX as compared wth conventional CAR T cells; these transcription factors were identified to be instrumental in initiating and maintaining T cell exhaustion in response to repetitive antigen stimulation.[27–29] The metabolism of those cells was superior to conventional CAR T cells at baseline and more substantially differed following repetitive antigen engagement. The superiority is based on glycolytic activities and augmented mitochondrial functions. In aggregate, we assume that increased CD39 after T cell activation poses a metabolic checkpoint limiting CAR T cell functionality upon prolonged antigen exposure. Consequently, the decrease of CD39 in CAR T cells maintains CAR T cell functionality during repetitive antigen stimulation by increasing metabolic rate, likely through reduced extracellular ATP degradation, and hence, inferred diminished adenosine-mediated suppression, and by keeping BLIMP-1, IRF-4, and TOX at low levels, all contributing to counteract T cell exhaustion. While recent lines of evidence imply the involvement of CD39 in T cell apoptosis, T cell exhaustion, and metabolic impairments,[16,18,19] our data support liberating CAR T cells from the repercussion through CD39 downregulation. At the single-cell level, CD39 downregulation was associated with enhanced cytotoxic competence, as reflected by increased CD107a surface exposure and elevated granzyme B expression in CD8^{+} CAR T cells following repeated antigen encounter. These findings suggest that improved antitumor activity is not solely attributable to differences in population expansion or survival but also involves preservation of effector function within individual CAR T cells under conditions of chronic stimulation. However, these analyses were performed in the context of a repetitive stimulation assay, in which antigen re-exposure, cell expansion, and selective survival occur in a tightly coupled manner. Consequently, this system does not fully resolve whether the observed increases in CD107a and granzyme B reflect sustained per-cell functionality, preferential retention of highly cytotoxic subpopulations, or shifts in population composition across stimulation rounds. Future studies employing strict normalization of effector-to-target ratios at each stimulation cycle, together with longitudinal single-cell tracking approaches, will be required to more precisely delineate these contributions and to disentangle intrinsic functional enhancement from selection-driven effects such as employed by Good et al.[30]

Improving CAR T cell resistance to exhaustion is a central endeavor aiming at improving CAR T cell efficacy. Numerous efforts were undertaken to target checkpoints on CAR T cells, such as PD-1, TIM3, LAG3, TIGIT, CD38, and CTLA4. Particularly, Wang et al. generated mesothelin-specific CAR T cells, in which, by means of CRISPR-Cas9 technology, both the endogenous T cell receptor and PD-1 expression were ablated.[10] In total, 15 patients were treated with PD-1-deficient CAR T cells, but no substantial tumor regression was attained, and CAR T cells showed poor persistence by less than one month,[10] whereas PD-1-deficient T cells expressing an NY-ESO-1-specific T cell receptor persisted for up to nine months in a clinical trial.[31] In line with these observations, preclinical data demonstrated a benefit of shRNA-mediated PD-1 knockdown for CAR T cells in animal models of prostate cancer and lymphoma.[32] Knockdown of TIM3 on mesothelin-specific CAR T cells using specific shRNA eventuated in enhanced cytotoxicity, cytokine secretion, and proliferative capacity of CAR T cells[11]; unfortunately, data on the functional CAR T cell persistence are lacking. In an effort to relieve CAR T cells from LAG3-mediated immunosuppression, Zhang et al. generated CAR T cells with abrogated LAG3 expression after CRISPR-Cas9-mediated gene knockout.[12] Despite robust silencing, LAG3-deficiency did not improve CAR T cell activity beyond the level of conventional CAR T cells concerning *in vitro* functionality and performance in a xenograft model.[12] While the knockout of every single checkpoint mostly remained mediocre, proof-of-principle data on the simultaneous knockdown of PD1, TIM3, and LAG3 in murine CAR T cells demonstrated superior survival and performance of CAR T cells in syngeneic and xenograft mouse models.[33,34] A comparative study revealed that only CAR T cells with PD-1/TIGIT double downregulation displayed improved *in vivo* efficacy.[14] While downregulation of PD-1/TIM-3 and PD-1/CTLA-4 did not improve CAR T cell functionality over PD-1 single knockdown, combined downregulation of PD-1 and LAG3 completely abrogated CAR T cell activity.[33] Recently, an optimized CAR T cell product was reported based on CRISPR-Cas9-mediated deletion of the checkpoint CTLA4, producing augmented CAR T cell proliferation and anti-tumor activity in preclinical models of blood cancer.[35] Similar to the results reported by Lee et al., a combined knockout of PD-1 and CTLA4 did not significantly improve CAR T cell activity, highlighting the benefit of selective CTLA4 deletion.[35] Collectively, preclinical and clinical efforts were mounted on investigating the knockdown of established immunosuppressive checkpoints on CAR T cells, such as PD-1, TIM3, LAG3, TIGIT, and CTLA4; however, durable improvement of CAR T cell functionality is still scant, spurring the quest to explore checkpoints on another level orchestrating T cell functionality. Our data indicate that CD39 downregulation is a valuable strategy to achieve durable improvement in T cell anti-cancer cell capacities. While studies on CD39-downregulated CAR T cells are still scarce, recently, CD39-deficient, HER-2-specific

CAR T cells were highlighted as a novel advanced cellular product for primary and metastatic colorectal cancer.[36] First, tissue specimens of patients with primary and metastatic colorectal tumors were analyzed by high-dimensional flow cytometry, RNA sequencing, and immunohistochemistry to establish that tumor-infiltrating T cells expressed a variety of inhibitory receptors. Second, CD39 was identified to be instrumental in driving T cell exhaustion in colorectal tumors.[36] Consequently, CRISPR-Cas9-genome-edited CD39 CAR T cells showed a functional advantage over conventional CAR T cells in an array of *in vitro* assays and *in vivo* studies involving patient-derived colorectal cancer organoids.[36] Nevertheless, the mechanistic basis for the superior functionality of T cells with CD39 downregulation was traced back to the immunosuppressive effects of adenosine but remains overall elusive; no information on metabolic changes was provided. The immunosuppressive effects of adenosine on CAR T cells are substantial, as the combination of CAR T cells with the selective adenosine A2A and A2B receptor antagonist AB928/etrumadenant[37] improved CAR T cell cytokine production, proliferation, and cytotoxicity *in vitro*. In this line, orally administered AB928/etrumadenant boosted CAR T cell activation in a syngeneic mouse model of colon carcinoma.[37] While blockade of adenosine A2A and A2B receptors efficiently shields from adenosine-induced suppression of T cell functionality, ATP depletion remains unaccounted for, although it represents the secondary immunosuppressive effect of CD39, causing metabolic stress and apoptosis in T cells.

Several observations in the present study, including improved metabolic fitness, reduced apoptosis, diminished expression of exhaustion-associated transcription factors, and enhanced anti-tumor activity, occurred in parallel with CD39 downregulation. While these findings support a model in which altered extracellular nucleotide metabolism contributes to sustained CAR T cell functionality, direct causal relationships between individual components of this network were not investigated. Furthermore, intracellular ATP concentrations were not directly measured. Therefore, the observed metabolic and transcriptional changes should be interpreted as being associated with reduced CD39 activity rather than as evidence for a fully defined mechanistic pathway. Future studies will be required to determine how extracellular ATP availability, adenosine signaling, metabolic programming, and exhaustion-associated transcriptional networks are mechanistically linked.

Our findings should be contextualized with prior studies investigating CD39 targeting in CAR T cells. So far, only few preclinical studies on CAR T cell products with synthetically decreased CD39 expression have been published. Klysz et al. evaluated the effect of CD39, CD73, or adenosine receptor 2a (A2aR) knockout in CAR T cells, and only modest effects on CAR T cell functionality were observed.[22] First, Klysz et al. employed an *in vitro* exhaustion model relying on artificial tonically signaling GD2-specific 4-1BB-costimulated CAR T cells (HA-CAR T cells), which usually display sign of T cell exhaustion within 10 days after activation. While the canonical exhaustion markers PD-1, TIM-3, and LAG3 were strongly upregulated on HA-CAR T cells, CD39 was only gradually upregulated, with a sizable proportion (around 25%) of HA-CAR T cells remaining CD39-negative. On conventional CD19-specifc CAR T cells, by contrast, no tangible upregulation of CD39 was observed. With regard to functionality, knockout of CD39 in HA-CAR T cells augmented IL-2 secretion but did not improve CAR T cell cytotoxicity. Importantly, IL-2 secretion of conventional CD19-CAR T cells was still superior as compared with HA-CAR T cells with CD39-knockout. Finally, Klysz et al. conducted transcriptomic analyses demonstrating an upregulation of the expression of stemness-associated genes in HA-CAR T cells with CD39-knockout, which, however, coincided with an unexpected decrease in the expression of genes governing CAR T cell functionality, such as granzym B and IFNγ.[22] In light of those findings, the approach of CD39 downregulation to boost CAR T cell functionality was discarded by Klysz et al., favoring a strategy based on exposure of CAR T cells to inosine during manufacturing. Nevertheless, the phenotype of CAR T cells after manufacturing remains plastic and is susceptible to reprogramming within the tumor microenvironment, especially in solid tumors such as in pancreatic cancer. In contrast to the observation by Klysz et al., we observed enhanced functionality, superior metabolic porperties, and decreased exhaustion markers in conjunction with a predominant effector memory phenotype. Moreover, differences could further originate from the selection of CAR constructs (aGD2-41BB-zeta versus aCEA-CD28-zeta). In addition, Zhang et al. reported enhanced antitumor activity of mesothelin-directed CAR T cells engineered to secrete a CD39-targeting nanobody. While these data support the therapeutic relevance of CD39, the relative contribution of CD39 inhibition on CAR T cells versus other CD39-expressing populations within the tumor microenvironment remained unresolved.[23] Because the nanobody was secreted into the extracellular space, both CAR T cell-intrinsic and extrinsic mechanisms may have contributed to the observed therapeutic benefit. Our approach differs conceptually by selectively reducing CD39 expression within the CAR T cell itself. This design enabled us to directly assess the consequences of CAR T cell-intrinsic CD39 modulation without broadly targeting other immune or stromal cell populations. Notably, CAR T cell activation induced substantial upregulation of CD39, and sustained CD39 downregulation was associated with improved metabolic fitness, reduced apoptosis, decreased expression of exhaustion-associated transcription factors, and superior long-term antitumor activity. These observations support the concept that CD39 functions as an activation-induced intrinsic metabolic checkpoint in CAR T cells. In aggregate, these studies, coupled with our data, suggest that while CD39 modulation can contribute to improved function, its effects are generally modest and context-dependent.

A limitation of the present study is that intracellular ATP concentrations were not directly measured. While CD39 downregulation clearly reduced extracellular ATP degradation and adenosine generation and was associated with improved glycolytic and mitochondrial function, the extent to which intracellular ATP pools are preserved remains to be determined. Therefore, the observed metabolic benefits are most

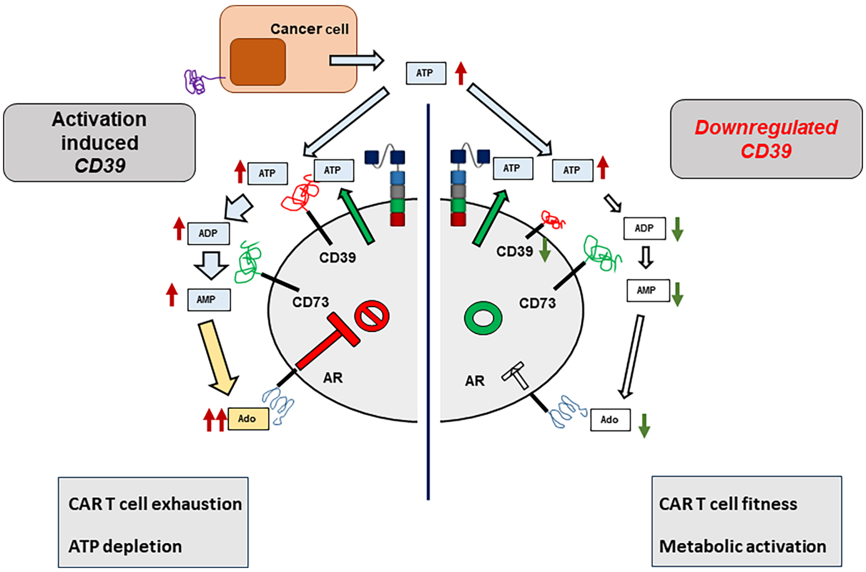

Figure 5. Proposed model of how CD39 downregulation may influence extracellular nucleotide metabolism, metabolic fitness, and CAR T cell persistence

Extracellular ATP, released from activated CAR T cells and dying tumor cells, is sequentially degraded by CD39 and CD73, resulting in adenosine generation. Adenosine can suppress T cell activation through adenosine receptor signaling. In the present study, CD39 downregulation reduced extracellular ATP degradation, decreased adenosine generation, improved metabolic fitness, reduced expression of exhaustion-associated markers, and enhanced CAR T cell persistence and antitumor activity.

appropriately interpreted as consequences of altered extracellular nucleotide metabolism and purinergic signaling rather than direct evidence of intracellular ATP preservation. Another limitation of our study is that the detailed functional and *in vivo* analyses were performed using a single CD39-targeting shRNA construct. Although four independent shRNAs were initially evaluated and all targeted the same gene, CEA-28ζ-CD39-1 was selected because it consistently produced the strongest and most durable reduction of CD39 expression following CAR stimulation. While the broad range of observed phenotypic, metabolic, and functional effects supports an on-target biological effect, we cannot formally exclude potential construct-specific contributions. Future studies employing additional CD39-targeting sequences, genetic knockout approaches, or rescue experiments will be valuable for further validating the mechanistic role of CD39 in CAR T cell biology.

In conclusion, we present evidence that CD39 downregulation is associated with enhanced metabolic fitness, reduced exhaustion, and improved functional persistence of CAR T cells under conditions of prolonged antigen engagement. Our findings support a model in which modulation of extracellular nucleotide metabolism contributes to these effects and provide a rationale for further mechanistic investigation of the pathways linking CD39 activity, metabolic programming, and CAR T cell durability (Figure 5). Additionally, we highlight CD39 downregulation as a strategy translating CAR T cells into having superior functionality during repetitive antigen encounter. Finally, the one-vector system, accommodating both the CD39-specific shRNA and the CAR, facilitates efficient GMP-compliant manufacturing of CAR T cells for future clinical evaluation. This is in line with our view of CD39 downregulation as one component of a broader strategy to enhance CAR T cell fitness, which may be most effective when combined with complementary interventions targeting additional metabolic, immunosuppressive, or exhaustion-associated pathways.

METHODS

Cells and reagents

Peripheral blood mononuclear cells (PBMCs) were isolated by Lymphoprep centrifugation (Axis-Shield, Oslo, Norway) from blood of healthy donors upon informed consent and approval by the institutional review board. PBMCs were cryopreserved and stored at −80°C until experimental use. T cells were cultured in RPMI 1640 medium, 1% (w/v) GlutaMAX (Gibco, ThermoFisher, Waltham, MA, USA), 100 IU/mL penicillin, 100 μg/mL streptomycin (Pan-Biotech, Aidenbach, Germany), 2 mM HEPES (PAA, Palo Alto, CA, USA), and 10% (v/v) heat-inactivated fetal calf serum (FCS) (Pan-Biotech, Aidenbach, Germany). 293T cells (ATCC CRL-3216 American Type Culture Collection, Manassas, VA) and BxPC-3 cells (ATCC CRL-1420) were cultured in DMEM, 1% (w/v) GlutaMAX (Gibco, ThermoFisher), 100 IU/mL penicillin, 100 μg/mL streptomycin (Pan-Biotech), and 10% (v/v) heat-inactivated FCS (Sigma-Aldrich, St. Louis, USA).

CAR T cell generation

Cryopreserved PBMCs were defrosted and activated on the same day with the anti-CD3 monoclonal antibody (mAb) OKT-3, the CD28 mAb 15E8, and IL-2 (1,000 IU/mL). Recombinant IL-2 (200 IU/mL) was added on days 2, 3, and 4 after activation without performing medium exchange. Retroviral transduction was performed as previously described.[26] Four days after activation, CAR T cells were purified via MACS, as previously described.[26] The CEA-specific CAR BW431/26scFv-Fc-CD28-ζ-K expression cassette was previously published.[24] The vectors encoding the CEA-specific CAR together with shRNAs targeting CD39 (TRCN0000050291, TRCN0000257193, TRCN0000363195, TRCN0000219070, TRCN0000230634, and TRCN0000230633), TIM3 (TRCN0000158033), LAG3 (TRCN0000157983), PD-1 (TRCN0000417811), and CD38 (TRCN0000050868) were synthesized by GenScript Biotech (Piscataway, N.J., USA).

Flow cytometry

Cells were stained with antibodies at 4°C for 15 min. For intracellular staining, cells were prepared using the transcription buffer set

(BD Biosciences, Franklin Lakes, NJ, USA) for 30 min at 4°C. The viability dye eFluor 780 (ThermoFisher, Waltham, MA, USA) was used for live/dead discrimination. Fluorescent-minus-one (FMO) controls were employed for gating. The goat F(ab')2 anti-human IgG-PE antibody and the goat F(ab')2 anti-human IgG-FITC antibody to detect the CAR were purchased from SouthernBiotech. The following antibodies were purchased from Miltenyi Biotech: FITC-conjugated anti-CD3 (clone BW 264/56), FITC-conjugated anti-CD8 (clone BW135/80), APC-conjugated anti-CD4 (clone VIT4), PE-conjugated anti-CD25 (clone 4E3), APC-conjugated anti-Granzyme B (clone REA 226), and PE-conjugated anti-CD73 (clone REA 804). The following antibodies were acquired from Biolegend (San Diego, CA, USA): PE-conjugated anti-CD38 (clone S17015A), BV421-conjugated anti-CD8 (clone RPA-T8), PE-conjugated anti-CD39 (clone A1), PerCP-Cy5.5-conjugated anti-CD27 (clone M-T271), PerCP-Cy5.5-conjugated anti-TIGIT (clone A15153G), BV421-conjugated anti-CD3 (clone OKT3), and PE-conjugated anti-IRF4 (clone IRF4.3E4) together with the corresponding isotype control antibody. The following antibodies were bought from BD Biosciences: BV421-conjugated anti-TIM3 (clone 7D3), BV421-conjugated anti-CD62L (clone DREG-56), BV605-conjugated anti-CD45RO (clone UCHL1), PE-conjugated anti-Blimp-1 (clone 6D3), BV421-conjuagted anti-CD137 (clone 4B4-1), BV421-conjugated anti-Ki67 (clone Ki-67), and BV421-conjugated anti-perforin (clone δG9). The following antibodies were purchased from ThermoFisher: PE-conjugated anti-TOX (clone TXRX10). For Annexin V staining, the PE Annexin V Apoptosis Detection Kit (BD Biosciences) was used according to the instructions of the manufacturer. For proliferation analysis, CAR T cells were labeled with 10 μM Cell Proliferation Dye eFluor 450 (ThermoFisher) before stimulation. Immunofluorescence was measured using a BD FACSLyric (BD Biosciences). Data were analyzed using the FlowJo software version 10.7.1 Express 5 (BD Biosciences).

ATP detection assay

Extracellular ATP in serum-free medium or cell culture supernatants was quantified employing the ATP Bioluminescence Assay Kit CLS II (Roche, Germany) according to the manufacturer's instructions. Supplemented ATP (Sigma) was dissolved in double-distilled water and stored at −80°C.

Adenosine detection assay

Extracellular adenosine in serum-free medium was quantified employing the Adenosine Quantification Assay Kit (Sigma-Aldrich) according to the manufacturer's instructions. Supplemented ATP (Sigma) was dissolved in double-distilled water and stored at −80°C.

Cytotoxicity assay

CAR T cells ($0.125–10 \times 10^4$ cells/well) were co-cultivated for 24 h in 96-well-round-bottom plates with target cells (1×10^4 cells/well) at the indicated effector to target ratios. The XTT-based colorimetric assay employing the Cell Proliferation Kit II (Roche Diagnostics, Mannheim, Germany) was used to analyze specific cytotoxicity. The percentage of viable tumor cells in experimental wells was determined as follows: viability (%) = (OD[experimental wells - corresponding number of T cells])/(OD[tumor cells without T cells - medium]) × 100. Cytotoxicity (%) was defined as 100 - viability (%).

Cytokine secretion

Target cells were maintained in 96-well-round-bottom plates (1×10^5 cells/well) overnight before adding CAR T cells (1×10^5 cells/well). Following 48 h of co-culture, IL-2 and IFN-γ in culture supernatants were determined by ELISA, as previously described.[38]

Repetitive stimulation assay

GFP-engineered BxPC-3 cells were seeded in 12-well plates (0.1×10^6 cells per well). After 24 h, 0.1×10^6 CAR T cells were added per well. Upon three days (round 1, R1), the wells were harvested and resuspended in 1 mL T cell medium. 100 μL was used for cell counting (live GFP^+ tumor cells and live $CD3^+$/CAR^+ CAR T cells) via flow cytometry using counting beads (CountBright, ThermoFisher). The remaining 900 μL was added to a new 12-well plate with 0.1×10^6 BxPC-3 cells for four days (round 2, R2). The procedure was reiterated for round 3 (R3) or round 4 (R4). For flow cytometric analyses, unlabeled BxPC-3 cells were employed.

Degranulation assay

At the end of round three of repetitive stimulation, CAR T cells were re-stimulated with BxPC-3 cells (seeded overnight in 96-well plates at 0.05×10^6 cells per well). Monensin (eBioscience, San Diego, CA, USA) at a final concentration of 1 μM and a FITC-conjugated anti-CD107a antibody (BD Biosciences, San Jose, CA, USA, clone: H4A3) were added at the beginning of stimulation. After four hours, T cells were stained with the viability dye eFluor 780 and a BV421-conjugated anti-CD8 antibody. $CD107a^+$ cells detected by flow cytometry indicated degranulated cells.

Seahorse metabolic assay

CAR T cells were resuspended in in Agilent Seahorse XF assay media with either (1) Glutmax, pyruvate, and glucose, no FCS (for Mito stress test) or (2) with Glutmax, no glucose, no FCS (for glycolysis stress test) and were seeded onto Seahorse cell culture microplates pre-coated with poly-L-lysine (50 μg/mL) at a seeding density of 200,000 cells per well (n = 3–5 replicates). The cell culture micro plate was spun at 300 ×g for 1 min to allow cell attachment and placed into a 37°C non-CO_2 incubator for 45 min prior to the assay. Drugs were prepared from a Seahorse XF Mito Stress Test Kit and Seahorse XF Glycolysis Stress Test Kit purchased from Agilent (Agilent, Santa Clara, CA, USA) and were loaded into the respective ports on the cartridges as per the manufacturer's instructions and run on a Seahorse XFe96 Analyzer. Glycolytic reserve was calculated by subtracting the basal glycolysis rate from the maximal glycolytic capacity. Spare respiratory capacity was calculated by subtracting the basal oxygen consumption rate from the maximal oxygen consumption rate.

Animal experiments

Female 7-week-old NSG mice (NOD.Cg-Prkdc ˆscid/Il2r ˆgtm1Wjl/SzJ) were obtained from the Jackson Laboratory and housed under specific pathogen-free conditions. Power analysis was conducted to determine the appropriate sample size. Mice were acclimatized for 7 days to their new environment and handling procedures. Random allocation to experimental groups was performed using a random number generator to ensure unbiased distribution. On day 0, each mouse received subcutaneous injections of 1×10^6 CEA^+ N87 gastric cancer cells expressing firefly luciferase (in 100 μL PBS) mixed with an equal volume of Matrigel (BD Biosciences, San Jose, CA, USA) in both flanks, resulting in two tumors per mouse. Each experimental group consisted of 4 mice (8 tumors per group). Kaplan-Meier survival analyses were performed at the tumor level; each tumor was treated as an individual experimental unit, while humane endpoint criteria requiring euthanasia of an animal resulted in simultaneous censoring of both tumors from that mouse. This experimental design was implemented in accordance with the 3Rs principles to reduce the number of animals used while enabling assessment of treatment effects across multiple tumor sites per biological replicate. Tumor growth was monitored by blinded personnel using an IVIS Spectrum CT system (PerkinElmer, Waltham, MA, USA). Prior to imaging, animals were anesthetized with isoflurane and injected intraperitoneally with D-luciferin (150 mg/kg). After 10 min, bioluminescence images were captured, and signal intensity was quantified using Living Image software (v.4.0, Caliper Life Sciences, Waltham, MA, USA). Signal intensity was expressed as total photons per second per square centimeter per steradian ($p/[s \times cm^2 \times sr]$) from identically sized regions of interest (ROIs). On day 10, each mouse was intravenously administered a single dose of 0.25×10^6 CAR T cells (CEA-28ζ-K as control; CEA-28ζ-CD39-1). An untreated control group (no T cell transfer) was included. Endpoint criteria for animal euthanasia were based on ethical guidelines and veterinary advice, which were met when animals exhibited signs of severe distress, pain, or suffering that could not be alleviated. No animals or data points were excluded from the analysis.

Statistical analysis

Statistical analysis was performed with GraphPad Prism, v.9 (GraphPad Software, San Diego, CA, USA). p values were determined as described in the figure legends.

DATA AND CODE AVAILABILITY

Original datasets are available from the corresponding author on reasonable request.

ACKNOWLEDGMENTS

The authors would like to thank Charlotte Schenkel for excellent technical assistance. This study was funded by the Deutsche Forschungsgemeinschaft (DFG, German Research Foundation) (grant no. 324392634—TRR221 to D.C.H.), the Else Kroner-Fresenius Foundation (to D.C.H.), the Helga Reifert Foundation, and the Wilhelm Sander Foundation (grant no. 2024.070.1 to D.C.H.). In addition, J.B. was supported by the Joachim Herz Foundation (add-on fellowship), and G.V. was supported by NKFIH OTKA K143771.

AUTHOR CONTRIBUTIONS

The concept of the study was designed by D.C.H., W.H., and H.A.; experimental design was done by D.C.H., H.P., M.B., G.V., and H.A.; experiments were performed by D.C.H., J.B., H.P., B.G., A.S., and M.B.; D.C.H. and H.A. wrote the manuscript. The manuscript was reviewed by all co-authors. All authors approved the submitted version.

DECLARATION OF INTERESTS

The authors declare no competing interests.

SUPPLEMENTAL INFORMATION

Supplemental information can be found online at https://doi.org/10.1016/j.omton.2026.201277.

REFERENCES

1. June, C.H., and Sadelain, M. (2018). Chimeric Antigen Receptor Therapy. N. Engl. J. Med. *379*, 64–73. https://doi.org/10.1056/NEJMra1706169.
2. Holzinger, A., and Abken, H. (2022). Treatment with Living Drugs: Pharmaceutical Aspects of CAR T Cells. Pharmacology *107*, 446–463. https://doi.org/10.1159/000525052.
3. Ormhøj, M., Abken, H., and Hadrup, S.R. (2022). Engineering T-cells with chimeric antigen receptors to combat hematological cancers: an update on clinical trials. Cancer Immunol. Immunother. *71*, 2301–2311. https://doi.org/10.1007/s00262-022-03163-y.
4. Schaft, N. (2020). The Landscape of CAR-T Cell Clinical Trials against Solid Tumors—A Comprehensive Overview. Cancers *12*, 2567. https://doi.org/10.3390/cancers12092567.
5. Safarzadeh Kozani, P., Safarzadeh Kozani, P., Ahmadi Najafabadi, M., Yousefi, F., Mirarefin, S.M.J., and Rahbarizadeh, F. (2022). Recent Advances in Solid Tumor CAR-T Cell Therapy: Driving Tumor Cells From Hero to Zero? Front. Immunol. *13*, 795164. https://doi.org/10.3389/fimmu.2022.795164.
6. Sterner, R.C., and Sterner, R.M. (2021). CAR-T cell therapy: current limitations and potential strategies. Blood Cancer J. *11*, 69. https://doi.org/10.1038/s41408-021-00459-7.
7. Blank, C.U., Haining, W.N., Held, W., Hogan, P.G., Kallies, A., Lugli, E., Lynn, R.C., Philip, M., Rao, A., Restifo, N.P., et al. (2019). Defining 'T cell exhaustion. Nat. Rev. Immunol. *19*, 665–674. https://doi.org/10.1038/s41577-019-0221-9.
8. Lynn, R.C., Weber, E.W., Sotillo, E., Gennert, D., Xu, P., Good, Z., Anbunathan, H., Lattin, J., Jones, R., Tieu, V., et al. (2019). c-Jun overexpression in CAR T cells induces exhaustion resistance. Nature *576*, 293–300. https://doi.org/10.1038/s41586-019-1805-z.
9. Thommen, D.S., and Schumacher, T.N. (2018). T Cell Dysfunction in Cancer. Cancer Cell *33*, 547–562. https://doi.org/10.1016/j.ccell.2018.03.012.
10. Wang, Z., Li, N., Feng, K., Chen, M., Zhang, Y., Liu, Y., Yang, Q., Nie, J., Tang, N., Zhang, X., et al. (2021). Phase I study of CAR-T cells with PD-1 and TCR disruption in mesothelin-positive solid tumors. Cell. Mol. Immunol. *18*, 2188–2198. https://doi.org/10.1038/s41423-021-00749-x.
11. Jafarzadeh, L., Masoumi, E., Mirzaei, H.R., Alishah, K., Fallah-Mehrjardi, K., Khakpoor-Koosheh, M., Rostamian, H., Noorbakhsh, F., and Hadjati, J. (2021). Targeted knockdown of Tim3 by short hairpin RNAs improves the function of anti-mesothelin CAR T cells. Mol. Immunol. *139*, 1–9. https://doi.org/10.1016/j.molimm.2021.06.007.
12. Zhang, Y., Zhang, X., Cheng, C., Mu, W., Liu, X., Li, N., Wei, X., Liu, X., Xia, C., and Wang, H. (2017). CRISPR-Cas9 mediated LAG-3 disruption in CAR-T cells. Front. Med. *11*, 554–562. https://doi.org/10.1007/s11684-017-0543-6.
13. Veliz, K., Shen, F., Shestova, O., Shestov, M., Shestov, A., Sleiman, S., Hansen, T., O'Connor, R.S., and Gill, S. (2024). Deletion of CD38 enhances CD19 chimeric antigen receptor T cell function. Mol. Ther. Oncol. *32*, 200819. https://doi.org/10.1016/j.omton.2024.200819.

14. Lee, Y.-H., Lee, H.J., Kim, H.C., Lee, Y., Nam, S.K., Hupperetz, C., Ma, J.S.Y., Wang, X., Singer, O., Kim, W.S., et al. (2022). PD-1 and TIGIT downregulation distinctly affect the effector and early memory phenotypes of CD19-targeting CAR T cells. Mol. Ther. *30*, 579–592. https://doi.org/10.1016/j.ymthe.2021.10.004.

15. Timperi, E., and Barnaba, V. (2021). CD39 Regulation and Functions in T Cells. IJMS *22*, 8068. https://doi.org/10.3390/ijms22158068.

16. Chow, A., Uddin, F.Z., Liu, M., Dobrin, A., Nabet, B.Y., Mangarin, L., Lavin, Y., Rizvi, H., Tischfield, S.E., Quintanal-Villalonga, A., et al. (2023). The ectonucleotidase CD39 identifies tumor-reactive CD8+ T cells predictive of immune checkpoint blockade efficacy in human lung cancer. Immunity *56*, 93–106.e6. https://doi.org/10.1016/j.immuni.2022.12.001.

17. Fang, F., Yu, M., Cavanagh, M.M., Hutter Saunders, J., Qi, Q., Ye, Z., Le Saux, S., Sultan, W., Turgano, E., Dekker, C.L., et al. (2016). Expression of CD39 on Activated T Cells Impairs their Survival in Older Individuals. Cell Rep. *14*, 1218–1231. https://doi.org/10.1016/j.celrep.2016.01.002.

18. Canale, F.P., Ramello, M.C., Núñez, N., Furlan, C.L.A., Bossio, S.N., Serrán, M.G., Boari, J.T., Del Castillo, A., Ledesma, M., Sedlik, C., et al. (2018). CD39 Expression Defines Cell Exhaustion in Tumor-Infiltrating CD8+ T Cells. Cancer Res. *78*, 115–128. https://doi.org/10.1158/0008-5472.CAN-16-2684.

19. Vignali, P.D.A., DePeaux, K., Watson, M.J., Ye, C., Ford, B.R., Lontos, K., McGaa, N.K., Scharping, N.E., Menk, A.V., Robson, S.C., et al. (2023). Hypoxia drives CD39-dependent suppressor function in exhausted T cells to limit antitumor immunity. Nat. Immunol. *24*, 267–279. https://doi.org/10.1038/s41590-022-01379-9.

20. Perrot, I., Michaud, H.-A., Giraudon-Paoli, M., Augier, S., Docquier, A., Gros, L., Courtois, R., Déjou, C., Jecko, D., Becquart, O., et al. (2019). Blocking Antibodies Targeting the CD39/CD73 Immunosuppressive Pathway Unleash Immune Responses in Combination Cancer Therapies. Cell Rep. *27*, 2411–2425.e9. https://doi.org/10.1016/j.celrep.2019.04.091.

21. Li, X.-Y., Moesta, A.K., Xiao, C., Nakamura, K., Casey, M., Zhang, H., Madore, J., Lepletier, A., Aguilera, A.R., Sundarrajan, A., et al. (2019). Targeting CD39 in Cancer Reveals an Extracellular ATP- and Inflammasome-Driven Tumor Immunity. Cancer Discov. *9*, 1754–1773. https://doi.org/10.1158/2159-8290.CD-19-0541.

22. Klysz, D.D., Fowler, C., Malipatlolla, M., Stuani, L., Freitas, K.A., Chen, Y., Meier, S., Daniel, B., Sandor, K., Xu, P., et al. (2024). Inosine induces stemness features in CAR-T cells and enhances potency. Cancer Cell *42*, 266–282.e8. https://doi.org/10.1016/j.ccell.2024.01.002.

23. Zhang, Y.-C., Li, X.-Y., Deng, Q., Ge, Y.-J., Yi, R.-R., Wang, H.-J., Wang, J.-T., Zhou, H., Kong, X.-F., Liu, R.-J., et al. (2024). Development of a CD39 nanobody and its enhancement to chimeric antigen receptor T cells efficacy against ovarian cancer in preclinical studies. Theranostics *14*, 6249–6267. https://doi.org/10.7150/thno.97590.

24. Harrer, D.C., Bezler, V., Hartley, J., Herr, W., and Abken, H. (2023). IRF4 downregulation improves sensitivity and endurance of CAR T cell functional capacities. Front. Immunol. *14*, 1185618. https://doi.org/10.3389/fimmu.2023.1185618.

25. Zhao, Z., Condomines, M., van der Stegen, S.J.C., Perna, F., Kloss, C.C., Gunset, G., Plotkin, J., and Sadelain, M. (2015). Structural Design of Engineered Costimulation Determines Tumor Rejection Kinetics and Persistence of CAR T Cells. Cancer Cell *28*, 415–428. https://doi.org/10.1016/j.ccell.2015.09.004.

26. Harrer, D.C., Schenkel, C., Bezler, V., Kaljanac, M., Hartley, J., Barden, M., Pan, H., Holzinger, A., Herr, W., and Abken, H. (2022). CAR Triggered Release of Type-1 Interferon Limits CAR T-Cell Activities by an Artificial Negative Autocrine Loop. Cells *11*, 3839. https://doi.org/10.3390/cells11233839.

27. Rutishauser, R.L., Martins, G.A., Kalachikov, S., Chandele, A., Parish, I.A., Meffre, E., Jacob, J., Calame, K., and Kaech, S.M. (2009). Transcriptional Repressor Blimp-1 Promotes CD8+ T Cell Terminal Differentiation and Represses the Acquisition of Central Memory T Cell Properties. Immunity *31*, 296–308. https://doi.org/10.1016/j.immuni.2009.05.014.

28. Man, K., Gabriel, S.S., Liao, Y., Gloury, R., Preston, S., Henstridge, D.C., Pellegrini, M., Zehn, D., Berberich-Siebelt, F., Febbraio, M.A., et al. (2017). Transcription Factor IRF4 Promotes CD8+ T Cell Exhaustion and Limits the Development of Memory-like T Cells during Chronic Infection. Immunity *47*, 1129–1141.e5. https://doi.org/10.1016/j.immuni.2017.11.021.

29. Seo, H., Chen, J., González-Avalos, E., Samaniego-Castruita, D., Das, A., Wang, Y.H., López-Moyado, I.F., Georges, R.O., Zhang, W., Onodera, A., et al. (2019). TOX and TOX2 transcription factors cooperate with NR4A transcription factors to impose $CD8^+$ T cell exhaustion. Proc. Natl. Acad. Sci. USA *116*, 12410–12415. https://doi.org/10.1073/pnas.1905675116.

30. Good, C.R., Aznar, M.A., Kuramitsu, S., Samareh, P., Agarwal, S., Donahue, G., Ishiyama, K., Wellhausen, N., Rennels, A.K., Ma, Y., et al. (2021). An NK-like CAR T cell transition in CAR T cell dysfunction. Cell *184*, 6081–6100.e26. https://doi.org/10.1016/j.cell.2021.11.016.

31. Stadtmauer, E.A., Fraietta, J.A., Davis, M.M., Cohen, A.D., Weber, K.L., Lancaster, E., Mangan, P.A., Kulikovskaya, I., Gupta, M., Chen, F., et al. (2020). CRISPR-engineered T cells in patients with refractory cancer. Science *367*, eaba7365. https://doi.org/10.1126/science.aba7365.

32. Zhou, J.e., Yu, J., Wang, Y., Wang, H., Wang, J., Wang, Y., Yu, L., and Yan, Z. (2021). ShRNA-mediated silencing of PD-1 augments the efficacy of chimeric antigen receptor T cells on subcutaneous prostate and leukemia xenograft. Biomed. Pharmacother. *137*, 111339. https://doi.org/10.1016/j.biopha.2021.111339.

33. Zou, F., Lu, L., Liu, J., Xia, B., Zhang, W., Hu, Q., Liu, W., Zhang, Y., Lin, Y., Jing, S., et al. (2019). Engineered triple inhibitory receptor resistance improves anti-tumor CAR-T cell performance via CD56. Nat. Commun. *10*, 4109. https://doi.org/10.1038/s41467-019-11893-4.

34. Ciraolo, E., Althoff, S., Ruß, J., Rosnev, S., Butze, M., Pühl, M., Frentsch, M., Bullinger, L., and Na, I.-K. (2022). Simultaneous Genetic Ablation of PD-1, LAG-3, and TIM-3 in CD8 T Cells Delays Tumor Growth and Improves Survival Outcome. IJMS *23*, 3207. https://doi.org/10.3390/ijms23063207.

35. Agarwal, S., Aznar, M.A., Rech, A.J., Good, C.R., Kuramitsu, S., Da, T., Gohil, M., Chen, L., Hong, S.-J.A., Ravikumar, P., et al. (2023). Deletion of the inhibitory co-receptor CTLA-4 enhances and invigorates chimeric antigen receptor T cells. Immunity *56*, 2388–2407.e9. https://doi.org/10.1016/j.immuni.2023.09.001.

36. Potenza, A., Balestrieri, C., Spiga, M., Albarello, L., Pedica, F., Manfredi, F., Cianciotti, B.C., De Lalla, C., Botrugno, O.A., Faccani, C., et al. (2023). Revealing and harnessing CD39 for the treatment of colorectal cancer and liver metastases by engineered T cells. Gut *72*, 1887–1903. https://doi.org/10.1136/gutjnl-2022-328042.

37. Seifert, M., Benmebarek, M.-R., Briukhovetska, D., Märkl, F., Dörr, J., Cadilha, B.L., Jobst, J., Stock, S., Andreu-Sanz, D., Lorenzini, T., et al. (2022). Impact of the selective A2AR and A2BR dual antagonist AB928/etrumadenant on CAR T cell function. Br. J. Cancer *127*, 2175–2185. https://doi.org/10.1038/s41416-022-02013-z.

38. Hombach, A.A., Rappl, G., and Abken, H. (2019). Blocking CD30 on T Cells by a Dual Specific CAR for CD30 and Colon Cancer Antigens Improves the CAR T Cell Response against CD30− Tumors. Mol. Ther. *27*, 1825–1835. https://doi.org/10.1016/j.ymthe.2019.06.007.

OMTON, Volume *34*

Supplemental information

Augmenting CAR T cell functionality

and metabolism through CD39 downtuning

Dennis Christoph Harrer, Jeremy Baldwin, Markus Barden, Hong Pan, Bence Gergely, Árpád Szöőr, György Vereb, Wolfgang Herr, and Hinrich Abken

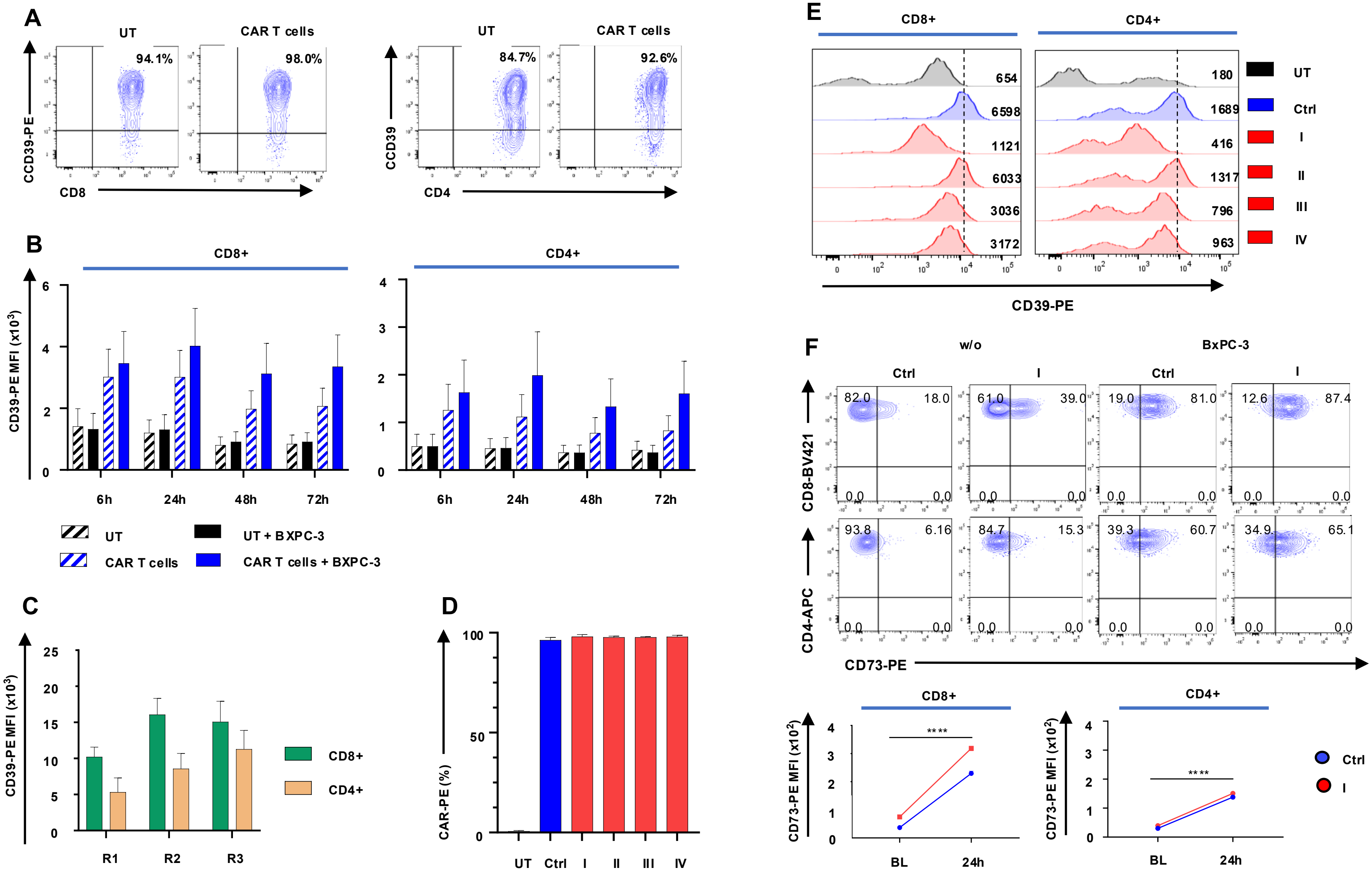

Figure S1: CD39 expression on CAR T cells. **(A)** CD39 expression on CD8⁺ and CD4⁺ CAR T cells was assessed by flow cytometry immediately after manufacturing (day 9 post-activation of PBMCs and retroviral transduction). Untransduced T cells (UT) served as controls. **(B)** Kinetics of CD39 expression on CD8⁺ and CD4⁺ CAR T cells following antigen-specific stimulation with CEA⁺ BxPC-3 pancreatic cancer cells, analyzed at the indicated time points post-stimulation. **(C)** CD39 expression during repetitive antigen stimulation. CAR T cells were co-cultured with CEA⁺ BxPC-3 cells over three stimulation rounds (R1–R3), each lasting three days. CD39 expression in CD8⁺ and CD4⁺ CAR T cells was analyzed by flow cytometry at the end of each round. **(D)** CEA-specific CAR T cells (CEA-28ζ-K = ctrl; CEA-28ζ-CD39-1 to CD39-4 = I–IV) were generated via retroviral transduction. UT cells were obtained by PBMC activation and expansion in IL-2 without CAR transduction. Following magnetic cell separation (MACS), CAR surface expression was assessed using a phycoerythrin (PE)-labeled goat anti-human IgG antibody. The percentage of CAR⁺ cells post-enrichment is shown. Data represent mean ± SEM of at least three donors. **(E)** Representative histograms showing CD39 expression (PE fluorescence) in CD8⁺ (left) and CD4⁺ (right) CAR T cells and UT cells, 24 hours after stimulation with CEA⁺ BxPC-3 cells. Mean fluorescence intensity (MFI) values for CD39-PE staining are indicated. Representative data from one of three donors are shown. **(F)** Representative dot plots showing CD73 expression in CD8⁺ and CD4⁺ CAR T cells (CEA-28ζ-K = Ctrl; CEA-28ζ-CD39-1 = I) 24 hours after stimulation with CEA⁺ BxPC-3 cells. Representative data from one of three donors are shown. Below, MFI of CD73-PE at baseline (BL) and 24 hours after antigen-specific stimulation with CEA⁺ BxPC-3 pancreatic cancer cells in CD8⁺ (left) and CD4⁺ (right) CAR T cells. Data represent mean ± SEM of three donors. Statistical significance was calculated using two-way ANOVA. ****$p \leq 0.0001$.

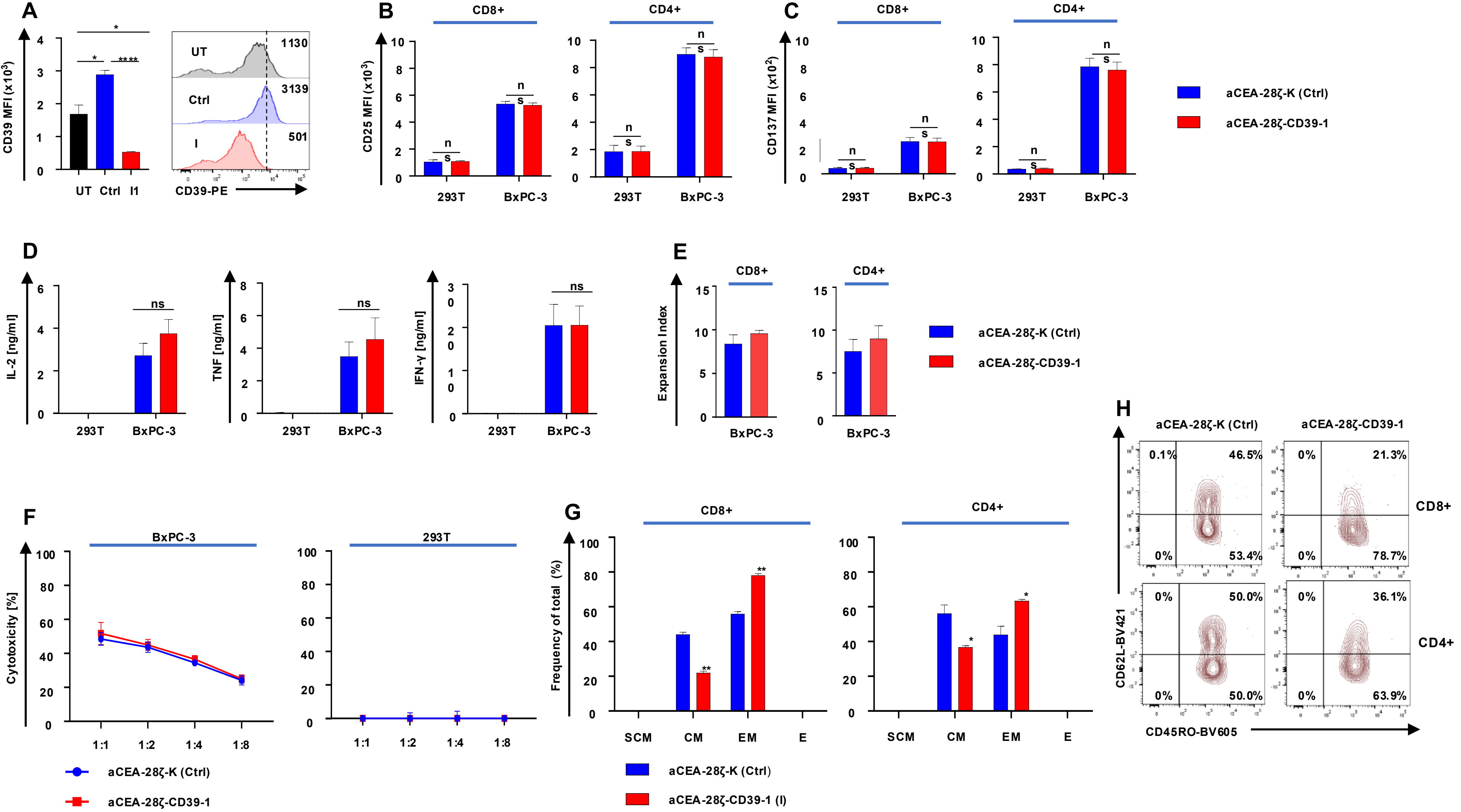

Figure S2: Impact of CD39 downregulation on basic CAR T cell effector functions. (A) CD39 expression in untransduced T cells (UT) and CAR T cells (CEA-28ζ-K = Ctrl; CEA-28ζ-CD39-1 = I) at the beginning of in vitro assays. CD39 expression was measured by flow cytometry, and mean fluorescence intensity (MFI) values of CD39-PE staining are shown in representative histograms. Data represent mean ± SEM of three donors. Statistical significance was determined using Student's t-test. *p ≤ 0.05; ***p ≤ 0.0001. (B) Antigen-specific upregulation of CD25 on CD8⁺ and CD4⁺ CAR T cells 24 hours after co-culture with CEA⁺ BxPC-3 pancreatic cancer cells or control 293T cells. (C) Antigen-specific upregulation of CD137 (4-1BB) on CD8⁺ and CD4⁺ CAR T cells 24 hours after co-culture with BxPC-3 or 293T cells. (D) Cytokine secretion (IL-2, TNF-α, IFN-γ) by CAR T cells after 48-hour co-culture with BxPC-3 or 293T cells, measured by ELISA. (E) Proliferative capacity of CD8⁺ and CD4⁺ CAR T cells after five-day stimulation with BxPC-3 cells. T cells were labeled with Cell Proliferation Dye eFluor® 450, and the expansion index was calculated based on dye dilution. (B–E) Data represent mean ± SEM of three independent donors. Statistical analysis was performed using Student's t-test. ns = not significant. (F) Cytotoxic activity of CAR T cells following a 24-hour co-culture with BxPC-3 or 293T target cells at the indicated effector-to-target (E:T) ratios. Cytotoxicity was measured using an XTT-based colorimetric assay. Data represent the mean ± SEM of four donors. (G) Differentiation of CAR T cells into effector memory subsets after three rounds (R1–R3) of antigen-specific stimulation with unlabeled BxPC-3 cells. Memory subsets were defined as follows: T stem cell memory (SCM, CD45RO⁺CD62L⁺), central memory (CM, CD45RO⁺CD62L⁺), effector memory (EM, CD45RO⁺CD62L⁻), and effector (E, CD45RO⁻CD62L⁻) cells. Data represent the mean ± SEM of three donors. Statistical significance was assessed by paired t-test. *p ≤ 0.05; **p ≤ 0.01. (H) Representative dot plots corresponding to Figure 2 G (three donors).

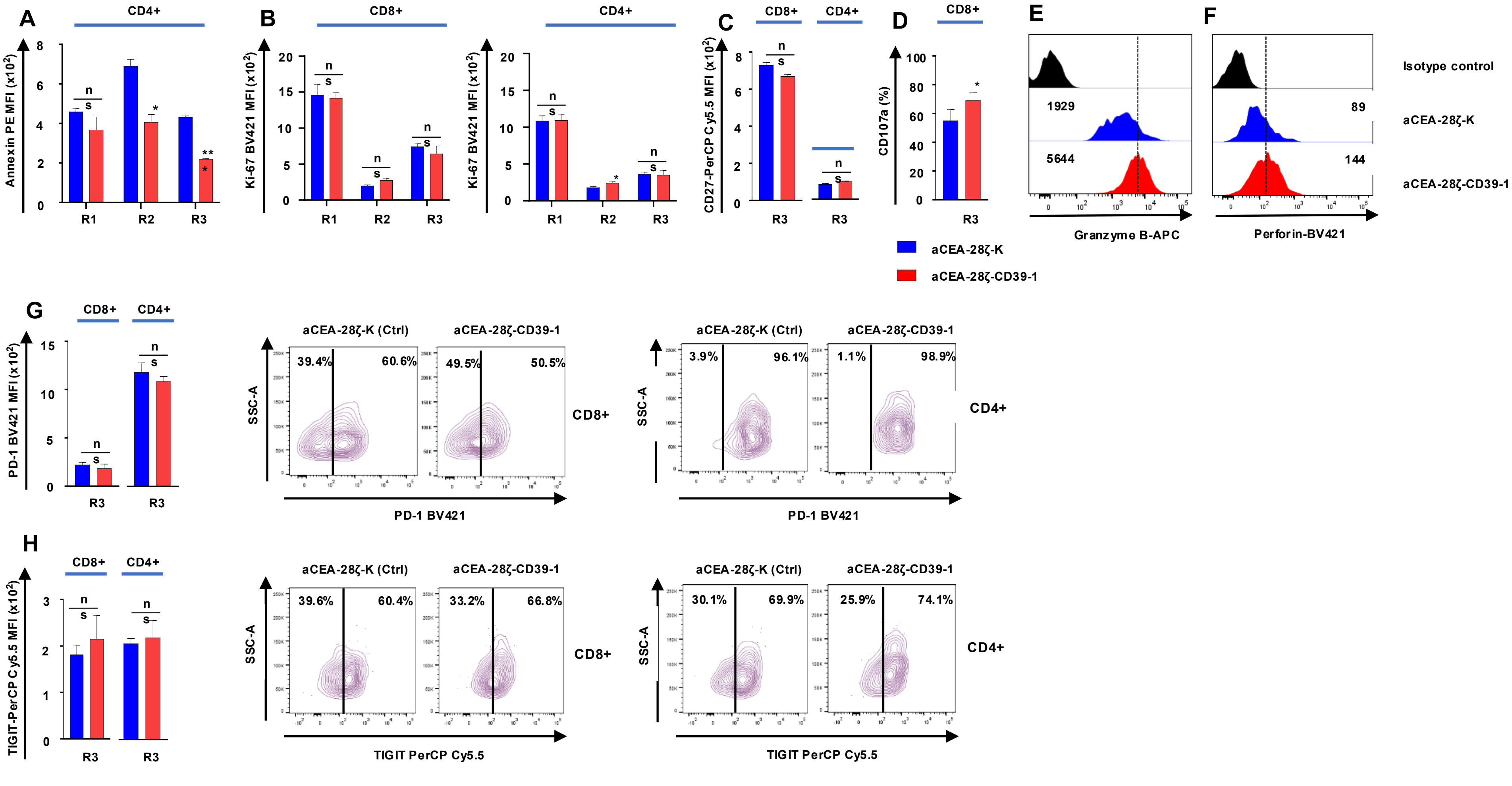

Figure S3: Phenotypic analysis of CAR T cells during repetitive antigen stimulation. CAR T cells (CEA-28ζ-K = ctrl; CEA-28ζ-CD39-1 = I) were stimulated three times (R1–R3) with unlabeled CEA$^+$ BxPC-3 pancreatic cancer cells. Functional and phenotypic markers were evaluated at the end of each round as indicated. **(A)** Apoptosis in CD4$^+$ CAR T cells was assessed at the end of each stimulation round by staining with Annexin V. **(B)** Proliferative capacity of CD8$^+$ and CD4$^+$ CAR T cells was measured by intracellular staining for Ki-67 at the end of each round. **(C)** Expression of CD27, a memory and survival marker, was evaluated in CD8$^+$ and CD4$^+$ CAR T cells at the end of round 3 (R3). **(D)** CD107a degranulation assay was performed on CD8$^+$ CAR T cells at the end of round 3 to assess cytotoxic potential. T cells were co-cultured with unlabeled BxPC-3 cells for 4 hours, and CD107a surface expression was measured by flow cytometry. **(E, F)** Expression of granzyme B **(E)** and perforin **(F)** in CD8$^+$ CAR T cells was analyzed at the end of round 3. Representative flow cytometry plots from one of three experiments are shown. **(G)** PD-1 expression in CD8$^+$ and CD4$^+$ CAR T cells at the end of round 3. **(H)** TIGIT expression in CD8$^+$ and CD4$^+$ CAR T cells at the end of round 3. **(A–D, G, H)** Data represent mean ± SEM of three independent donors or representative dot plots (three donors). Statistical analysis was performed using Student's t-test. *$p \leq 0.05$; ns = not significant.

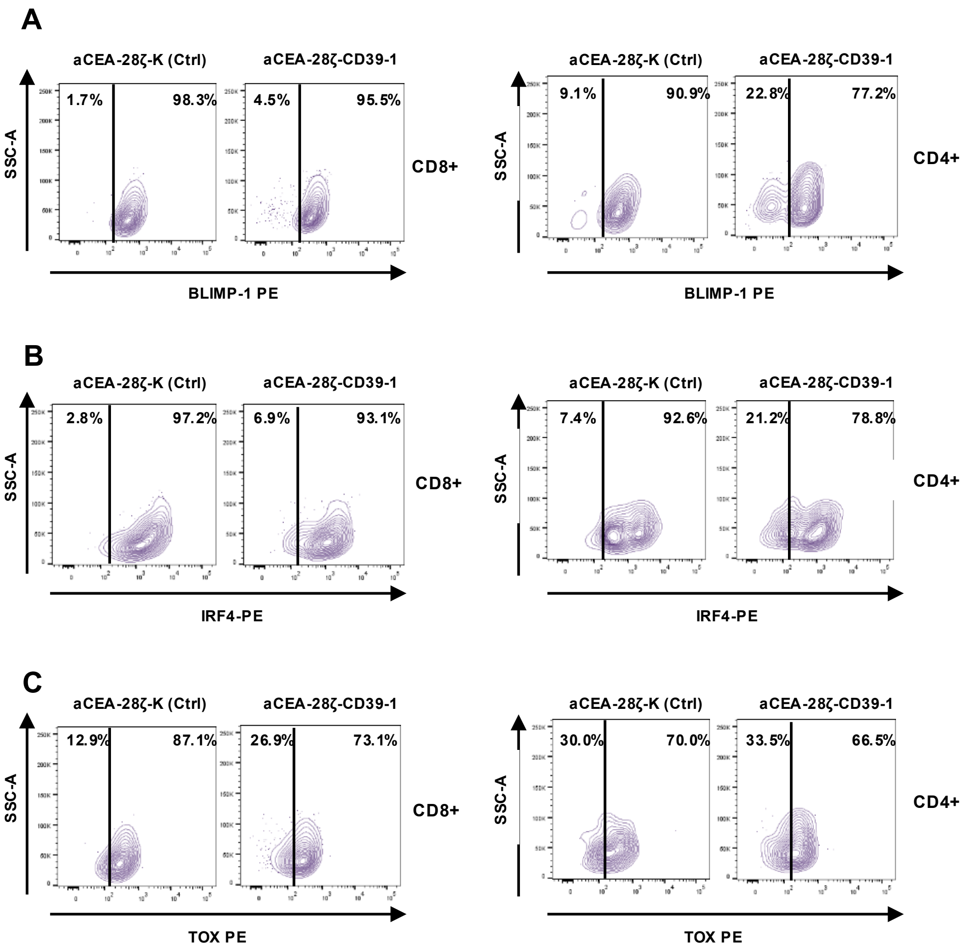

Figure S4: **Expression of exhaustion-related transcription factors in CAR T cells during repetitive antigen stimulation.** CAR T cells (CEA-28ζ-K = ctrl; CEA-28ζ-CD39-1 = I) were stimulated three times (R1–R3) with unlabeled CEA^{+} BxPC-3 pancreatic cancer cells. Expression of BLIMP-1 **(A)**, IRF-4 **(B)**, and TOX **(C)** was determined via flow cytometry at the end of round three. One representative dot plot out of three donors is depicted.

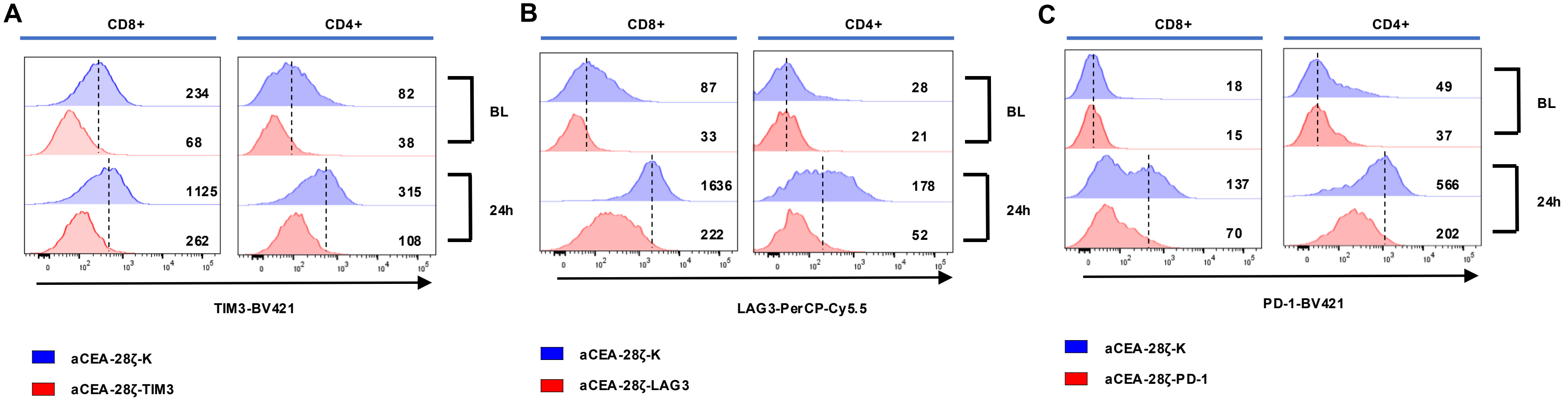

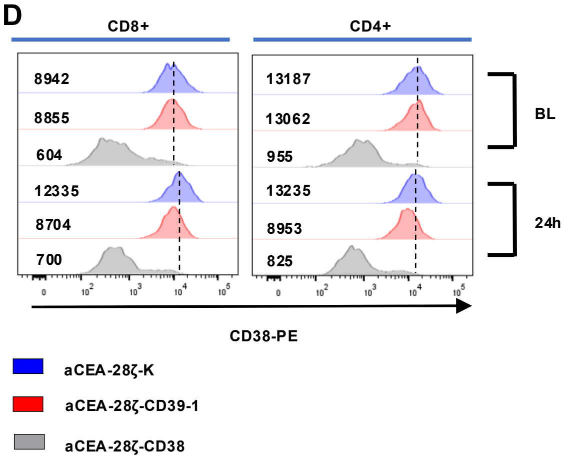

Figure S5: Downregulation of TIM-3, LAG-3, PD-1, and CD38 in CAR T Cells. (A–D) Representative histograms showing surface expression of inhibitory receptors at baseline (BL) and 24 hours after antigen-specific stimulation with CEA$^+$ BxPC-3 pancreatic cancer cells in CD8$^+$ (left) and CD4$^+$ (right) CAR T cells. **(A)** TIM3**. (B)** LAG3**. (C)** PD-1. **(D)** CD38. Mean fluorescence intensity (MFI) values are indicated in each histogram. Data shown are from one representative donor out of three independent experiments.
